# Review and evaluation of penalised regression methods for risk prediction in low‐dimensional data with few events

**DOI:** 10.1002/sim.6782

**Published:** 2015-10-29

**Authors:** Menelaos Pavlou, Gareth Ambler, Shaun Seaman, Maria De Iorio, Rumana Z Omar

**Affiliations:** ^1^ Department of Statistical ScienceUniversity College LondonLondonWC1E 6BTU.K.; ^2^ MRC Biostatistics UnitCambridgeCB2 0SRU.K.

**Keywords:** shrinkage, Bayesian regularisation, overfitting, rare events

## Abstract

Risk prediction models are used to predict a clinical outcome for patients using a set of predictors. We focus on predicting low‐dimensional binary outcomes typically arising in epidemiology, health services and public health research where logistic regression is commonly used. When the number of events is small compared with the number of regression coefficients, model overfitting can be a serious problem. An overfitted model tends to demonstrate poor predictive accuracy when applied to new data. We review frequentist and Bayesian shrinkage methods that may alleviate overfitting by shrinking the regression coefficients towards zero (some methods can also provide more parsimonious models by omitting some predictors). We evaluated their predictive performance in comparison with maximum likelihood estimation using real and simulated data. The simulation study showed that maximum likelihood estimation tends to produce overfitted models with poor predictive performance in scenarios with few events, and penalised methods can offer improvement. Ridge regression performed well, except in scenarios with many noise predictors. Lasso performed better than ridge in scenarios with many noise predictors and worse in the presence of correlated predictors. Elastic net, a hybrid of the two, performed well in all scenarios. Adaptive lasso and smoothly clipped absolute deviation performed best in scenarios with many noise predictors; in other scenarios, their performance was inferior to that of ridge and lasso. Bayesian approaches performed well when the hyperparameters for the priors were chosen carefully. Their use may aid variable selection, and they can be easily extended to clustered‐data settings and to incorporate external information. © 2015 The Authors. Statistics in Medicine Published by JohnWiley & Sons Ltd.

## 1 Introduction

The usefulness of risk prediction models for informing patients and practitioners about the future course of a disease, guiding therapeutic strategies, aiding selection of patients for inclusion in randomised trials and in surveillance has been well established 1, 2, 3. Often, a risk prediction model is developed using a regression model that associates the outcome to patient characteristics, the predictor variables. For binary outcomes, a logistic regression model is commonly used. In model fitting, the regression model is fitted to the data at hand (training or development data set) to estimate the regression coefficients. These estimated coefficients can then be used to predict the outcome in new patients. However, a risk model that performs well on the training data set may not perform equally well when it is applied to new data. Therefore, its predictive performance needs to be assessed, that is, the model needs to be validated before it can be used to make predictions. A good risk model should be able to demonstrate good calibration, discrimination and predictive accuracy in new data. Since last decade, risk models that are commonly used in practice such as the ‘QRISK‐2’ and the ‘Framingham’ calculator for the risk of cardiovascular disease 4, 5 and the ‘HCM‐SCD calculator’ for the risk of sudden cardiac death in patients with hypertrophic cardiomyopathy 6, all use the coefficients estimates from the model fitting directly into a risk calculator to calculate risks for patients. In this paper, we focus on this form of risk calculation.

There are several reasons why the apparent performance of a model might not hold in the validation data. One of these is model overfitting, which is likely to arise when the number of events in the training set is small compared with the number of estimated regression coefficients. In health research, such scenarios may arise, for example, in studies of rare events or rare diseases. An overfitted model tends to capture not only the underlying processes that generated the data but also noise in the training data set, and typically overestimates or underestimates the risk of the event for high‐risk or low‐risk patients. For example, a cardiac risk model has been developed to predict the 5‐year risk of sudden death (rare event) in patients with hypertrophic cardiomyopathy and guide the implantation of implantable cardioverter defibrillators (a small device used to regulate arrhythmias) 6. Among 3672 patients with complete data, there were only 42 sudden deaths within the first year after first evaluation. Overestimating the risk of sudden death could lead to the unnecessary implantation of implantable cardioverter defibrillators, exposing patients to unnecessary risks and also wasting resources. An example of a rare disease is penile cancer, which has an annual incidence of 1.3–2.0 per 100 000 men. In a study to predict the risk of death from penile cancer 7, only 128 patients with penile cancer were available of which 25 died, making the development of a reliable prediction model challenging.

Often, after employing expert knowledge to reduce the number of potential predictors, the ratio of the number of events to the number of regression coefficients to be estimated (‘events per variable’ or EPV) is low. As a rule of thumb, it has been suggested that prediction models are likely to be reliable when the EPV is at least 10 (e.g. 8). This cut‐off has been challenged as not being based on convincing scientific reasoning 3, and in fact, it is based on empirical evidence for obtaining valid estimates of standard errors for regression coefficients, rather than reliable risk prediction 9, 10. Nevertheless, the EPV is frequently mentioned in reports of clinical prediction studies, as revealed by the systematic review of Bouwmeester *et al.*, 11, and is useful for quantifying the amount of information in the data relative to model complexity. In order to meet the EPV recommendations, researchers employ practical approaches such as stepwise selection and univariable screening to reduce the EPV. Note that EPV should be based on the initial model, *before* any variable selection is carried out. These naive approaches have long been known to have significant drawbacks, and notes of caution have been raised 8, 12, 13. Additionally, it is not unusual that even after reducing the EPV by univariable screening or stepwise selection, the resulting model still has low EPV and, therefore, is susceptible to overfitting.

One simple way to reduce overfitting is to fit the model using maximum likelihood estimation (MLE) and then shrink the regression coefficients, post‐estimation, by a common factor (linear shrinkage factor (LSF)). The shrinkage factor is in essence an estimate of the amount of overfitting and can be obtained using bootstrapping 8. Harrell 8 noted that using an LSF is not as good as building shrinkage into the estimation process using penalised regression. The latter allows differential shrinkage of the regression coefficients. Two popular penalised regression methods are ridge and lasso. Both shrink the regression coefficients towards zero; lasso can also perform variable selection by shrinking some coefficients to exactly zero. For risk prediction in low‐dimensional settings, little attention has been devoted to these methods. Ambler *et al.*, 13 explored the use of ridge and lasso for low‐dimensional survival data with few events and concluded that both methods improve calibration, discrimination and predictive accuracy compared with standard Cox regression. They also used backwards elimination, LSF and univariable screening and found that these methods were inferior to ridge or lasso, with LSF tending to over‐shrink in low EPV scenarios. Steyerberg *et al.*, 12 suggested that shrinkage is necessary when *E* *P* *V* < 10 and advisable for 10 < *E* *P* *V* < 20. Extensions of ridge and lasso, such as elastic net, adaptive lasso and smoothly clipped absolute deviation (SCAD), have been proposed relatively recently. These have been proposed to improve variable selection compared with lasso and have been mainly used in high‐dimensional settings. Their predictive performance in low‐dimensional settings has been studied less. Porzelius *et al.*, 14 used bootstrapping to compare lasso, elastic net and SCAD in low‐dimensional survival data. They predominantly studied variable selection aspects when the EPV exceeded 10 and found that SCAD performed best when there were both true and noise predictors in the model. For EPV less than 10, they found that all techniques had lower prediction error than standard Cox regression.

As previously mentioned, some of the methods considered in this work (e.g. lasso) can also perform variable selection in the sense that in addition to shrinking regression coefficient estimates towards zero, they may also exclude ‘superfluous’ predictors by shrinking their coefficients to exactly zero. This is important when a more parsimonious model is sought. The various methods have different properties with respect to selecting true predictors with high probability or excluding noise predictors with high probability, as we discuss later. However, prediction and variable selection are two separate issues, and in this paper, we primarily focus on the first. So, we are interested in comparing different methods with respect to their ability to produce models with good predictive performance.

One challenge when using frequentist penalised methods is obtaining meaningful estimates of precision for the parameter estimates 15. Kyung *et al.*, 16 demonstrated the problem of obtaining a valid standard error for a coefficient whose true value is zero when lasso is used. They argued that using a Bayesian approach to shrinkage would provide valid precision measures (posterior standard deviations and credible intervals). It would also yield, for each coefficient, a posterior distribution, which could help with any variable selection decisions. It is important to note, however, that frequentist coverage of the posterior credible intervals is not guaranteed because the likelihood does not dominate the prior unless the sample size is large 16, 17.

Bayesian analogues of ridge and lasso are often referred to as ‘Bayesian regularisation’. They use normal and double exponential priors, respectively, for the regression coefficients. Another popular approach to performing Bayesian variable selection and estimation is based on ‘spike and slab’ priors, but its use for prediction in low‐dimensional data has been infrequent. From the wide spectrum of Bayesian methodology for estimation and variable selection, in this work, we focus on Bayesian regularisation and the spike and slab approach. Rockova *et al.*, 18 applied a large selection of Bayesian approaches in low‐dimensional settings and focused on the ability of the methods to select the right subset of potential predictors, rather than on the predictive performance of the derived models. They found that in sparse scenarios (i.e. scenarios with many noise predictors compared with the number of true predictors), the spike and slab approach performed best, while in non‐sparse scenarios, Bayesian regularisation was better.

In this article, we focus on the predictive performance of regression models with low‐dimensional data, binary outcome and few events. We (i) evaluate the predictive performance of frequentist penalised methods using simulation studies. We also investigate the sensitivity of the results to the choice of tuning parameters via a single cross‐validation and to the method of standardisation of predictors; (ii) explore the use of Bayesian approaches to alleviate overfitting; (iii) discuss and explore the ability of frequentist and Bayesian methods to construct confidence/credible intervals for predicted probabilities; and (iv) make recommendations about application of these methods in practice.

The article is organised as follows. In Section 2, we provide a review of frequentist and Bayesian shrinkage methods, listing their main theoretical properties relevant to risk prediction in low‐dimensional settings. Section 3 presents a case study where a real low‐EPV data set of patients with penile cancer is analysed using the various approaches. In Section 4, we simulate binary data based on the penile cancer data set for two EPV scenarios and compare the predictive performance of the methods. We also explore the coverage properties of confidence and credible intervals for predicted probabilities and discuss the bias introduced in the coefficient estimates for each of the methods considered. We consider further scenarios of interest in Section 5 and use artificial data to explore these. We present a data illustration in Section 6, and we conclude with a discussion and recommendations about the practical application of the methods.

## 2 Regression methods for predictive modelling in data sets with few events

### 2.1 Classical methods

For binary data, a logistic regression model is commonly used: logit(*E*(*Y*|***X***)) = ***β*** ^*T*^ ***X***, where *Y* is the binary outcome, ***X*** = (1,*X*_1_,…,*X*_*p*_) is the vector of covariate values and ***β*** = (*β*_0_,*β*_1_,…,*β*_*p*_)^*T*^ is a (*p* + 1)−dimensional vector of regression parameters. In MLE, the model is fitted by maximising the log‐likelihood function denoted by *l*(***β***). In penalised likelihood estimation, *l*(***β***) is instead maximised subject to constraints on the values of the regression coefficients. The regression coefficient estimates are typically shrunk towards zero in comparison with MLE, and this may help alleviate overfitting.

Often, penalised likelihood estimation is expressed as an optimisation problem of finding the value of ***β*** which, in the simplest case, maximises a function of the form *l*(***β***) − *λ*pen(***β***), where pen(***β***) is the ‘penalty term’ and *λ* is the ‘tuning’ parameter. The penalty term corresponds to the functional form of the constraint, and the tuning parameter corresponds to the amount of shrinkage applied; *λ* = 0 corresponds to standard MLE. Different forms for the penalty have been proposed (some of which may involve more than one tuning parameter). The tuning parameter is usually selected using a data‐driven procedure such as cross‐validation 19 to maximise the ‘out‐of‐sample’ performance of the model. We now consider the most popular types of penalised methods.

- Ridge 20, or *L*_2_ penalization, uses a penalty proportional to the sum of squares of regression coefficients, so $\hat{\beta }=\text{argmax}\left\{l(\beta )-\lambda_{2}\overset{p}{\underset{j=1}{\sum }}\beta_{j}^{2}\right\}$. Ridge was initially designed to deal with issues of multicollinearity 21. In the context of risk prediction, it shrinks the regression coefficients towards zero and has been seen to perform well in scenarios with correlated predictors.
- Lasso 22, or *L*_1_ penalization, imposes a constraint on the sum of the absolute value of regression coefficients, so $\hat{\beta }=\text{argmax}\left\{l(\beta )-\lambda_{1}\overset{p}{\underset{j=1}{\sum }}|\beta_{j}|\right\}$. It shrinks the regression coefficients towards zero but can also perform variable selection by shrinking some coefficients to exactly zero. This method has been seen to under‐perform in the case of correlated predictors in the sense that it may select one at random from a group of highly correlated predictors 23. This can affect the interpretability of the model and compromise its predictive accuracy 23. B $\ddot{\text{u}}$hlmann and van de Geer 24 explored the theoretical properties of lasso and noted that it should mostly be seen as a variable ‘screening’ rather than ‘selection’ method, in the sense that it tends to select large models by allowing some noise predictors to enter the model.
- ‘Elastic net’ 23, a hybrid of ridge and lasso, has a penalty with both ridge and lasso parts: $\hat{\beta }=\text{argmax}\left\{l(\beta )-\lambda_{1}\overset{p}{\underset{j=1}{\sum }}|\beta_{j}|-\lambda_{2}\overset{p}{\underset{j=1}{\sum }}\beta_{j}^{2}\right\}$. As such, it shares the strengths of the two: it can produce more parsimonious models than ridge by performing variable selection while also tending to select or omit highly correlated predictors as a group.
- Adaptive lasso 25 is another variant of lasso. It allows a different weight for each parameter in the penalty term: $\hat{\beta }=\text{argmax}\left\{l(\beta )-\lambda_{1}\overset{p}{\underset{j=1}{\sum }}\omega_{j}|\beta_{j}|\right\}$. The weights *ω*_*j*_ are data‐dependent (usually the inverse of the corresponding coefficient from ridge regression). As a result, coefficients of strong predictors are shrunk less than the coefficients of weak predictors. Zou 25 showed that when the weights are data‐dependent and appropriately chosen, the adaptive lasso can enjoy the oracle property (i.e. the probability that it correctly selects the predictors with non‐zero coefficients converges to one as the sample size increases and the estimators of the non‐zero coefficients are asymptotically normal with the same mean and covariance that they would have if the zero coefficients were known in advance). Adaptive lasso has been designed for variable selection in high‐dimensional settings, and Zou 25 noted that the oracle property does not guarantee optimal predictive performance in finite samples; instead, lasso can be better for certain prediction problems (for example when coefficients are small and/or the noise predictors are few 26, 27).
- Smoothly clipped absolute deviation (SCAD) 28 is another method that performs parameter estimation and simultaneous variable selection. It uses a non‐concave quadratic spline penalty function:
  $$\mathrm{pen}(\beta_{j})=\left\{\begin{array}{cc} \lambda |\beta_{j}| & \;\text{if}\;|\beta_{j}|<\lambda \\ \frac{\lambda (a-|\beta_{j}|/2\lambda )}{a-1} & \;\text{if}\;\lambda <|\beta_{j}|\leq \mathrm{a\lambda}\\ \lambda \frac{a^{2}\lambda }{2(a-1)|\beta_{j}|} & \;\text{if}\;|\beta_{j}|>\mathrm{a\lambda}. \end{array}\right.$$
  There are two tuning parameters to be chosen, *a* > 2 and *λ* > 0. Fan 2001 28 suggested using *a* = 3.7 and selecting *λ* using cross‐validation. In principle and in practice, SCAD applies a shrinkage pattern similar to that of adaptive lasso, so large coefficients are penalised less than small coefficients.

Predictors are standardised to have mean zero and unit variance before using penalised estimation. To obtain regression coefficient estimates on the original scale, each coefficient estimate is divided by the original standard deviation of the corresponding predictor. The intercept on the original scale is ${\hat{\beta }}_{0}^{\mathrm{or}}={\hat{\beta }}_{0}^{\mathrm{sc}}-\overset{p}{\underset{j=1}{\sum }}\bar{X_{j}}\;{\hat{\beta }}_{j}^{\mathrm{sc}}$, where the superscript *sc* indicates the coefficients for the standardised data. We adopted this scaling, which is the default option in most software packages, but other scaling patterns have also been proposed. For example, Gelman *et al.*, 29 suggested centering all predictors and scaling continuous predictors to have standard deviation of 0.5, equal to the standard deviation of a binary predictor with prevalence of 0.5, their reasoning mainly being to obtain more interpretable coefficient estimates on a ‘common’ scale. For the settings considered in this paper, there was no indication that the two scaling methods result in substantially different predictive performances of the methods (further discussion on the choice of standardisation can be found in the Supporting Information material, Section S2).

#### 2.1.1 Confidence intervals for regression coefficients and predicted probabilities

Confidence intervals for predicted probabilities can provide an indication of how precise the estimate is. In some situations in clinical practice, it may be useful to communicate to a patient a range for the predicted probability rather than a single point estimate. However, confidence intervals around estimates of regression coefficients and individual predicted probabilities obtained from penalised methods are problematic. Penalised methods introduce bias towards the null (to reduce the mean squared error), and confidence intervals are not very meaningful for strongly biased estimators 30. Most software packages deliberately do not provide standard errors for the coefficients. Although standard errors can be obtained using bootstrapping 22, Kyung *et al.*, 16 raise a note of caution regarding their use. In particular, they showed that for lasso, the bootstrap standard errors are not valid for the coefficients with zero true value. Chatterjee *et al.*, 31 and Sortari 15 suggested modified bootstrap methods, but their improvements mostly apply only in linear models. Despite these issues, if one wishes to calculate confidence intervals for the predicted probabilities, these may be obtained using the Delta method 32 after first using bootstrap to estimate var(***β***).

### 2.2 Bayesian regularisation and spike and slab priors

#### 2.2.1 Bayesian regularisation

The majority of the classical penalised methods have a Bayesian analogue, often referred to as ‘Bayesian Regularisation’. Most regularisation priors are, conditionally on a variance parameter, Gaussian: *β*_*j*_|*σ* ^2^∼*N*(0,*σ* ^2^). Different prior assumptions about *σ* ^2^ induce different marginal distributions for *β*_*j*_.

For Bayesian ridge regression, *β*_*j*_∼Normal(0,*σ* ^2^). If *σ* ^2^ is treated as fixed rather than assigned a prior, then *λ* = 1/*σ* ^2^ corresponds to the tuning parameter of the frequentist ridge. Bayesian and frequentist ridge are the same in the sense that the posterior mode (and mean) of the regression coefficients equal the estimates from frequentist ridge. To allow a more flexible shrinkage pattern of the regression coefficients, *σ* ^2^ can be assigned a suitable hyperprior, usually *σ* ^2^∼InvGamma(*a*,*b*), in which case marginally, the coefficients follow a scaled Student's *t* distribution.

In Bayesian lasso 33, the following hierarchical specification is often used: *β*_*j*_|*σ* ^2^∼Normal(0,*σ* ^2^); *σ* ^2^|*λ* ^2^∼Exp(0.5*λ* ^2^); *λ* ^2^∼Gamma(*a*,*b*). Then, *β*_*j*_ follows a Laplace (double exponential) distribution:*β*_*j*_|*λ* ∼ *D* *E*(*λ*) given *λ*. If *λ* is taken as fixed rather than assigned a gamma prior, then *λ* corresponds to the tuning parameter in the frequentist lasso, and the *mode* of the posterior distribution of the coefficients (‘Maximum a posteriori’ or ‘MAP’ estimates) coincides with the estimates of the traditional lasso. More often, a hyperprior is assigned to *λ* and variable selection is carried out by examining the posterior distribution of the regression coefficients. As the posterior mean/median is never zero with positive probability, variable selection is performed by ‘hard shrinkage’, that is, a coefficient is set to zero if the absolute value of its mean/median does not exceed a specified threshold.

Fahrmeir *et al.*, 34 noted that if we assign hyperpriors to the tuning parameters for Bayesian ridge and lasso, the interpretation of the penalty changes (compared with treating the tuning parameters as fixed). In particular, the marginal priors of Bayesian ridge and lasso are quite close after integrating out the hyperparameters, and the two tend to perform very similarly 35. In our real analyses and simulations, Bayesian lasso and Bayesian ridge indeed gave nearly identical estimates. We therefore present only results for the first. For Bayesian lasso, it is suggested that the Gamma hyperprior for *λ* ^2^ be chosen to be relatively flat 33.

#### 2.2.2 Estimation and variable selection based on spike and slab priors

An alternative Bayesian approach suitable for coefficient estimation while performing simultaneously variable selection is based on ‘spike and slab’ priors. Each component of ***β*** has a prior that is a mixture of a distribution with its mass concentrated around zero (‘spike’) and one with a mass spread over a large range of values (‘slab’).

George *et al.*, 36 used the ‘stochastic search variable selection’ approach (SSVS), where the spike and slab prior is of the form *β*_*j*_∼(1 − *γ*_*j*_)*N*(0,*σ* ^2^) + *γ*_*j*_ *N*(0,*c* ^2^ *σ* ^2^). The binary inclusion indicators, *γ*_*j*_, are assigned Bernoulli(*q*) prior distributions, and a suitable hyperprior is assigned to *q* (we use *q* ∼ Uniform[0,1], but other options e.g. a Beta distribution could also be used). The parameter *c* (*c* > 1), chosen by the analyst, expresses the difference between the spike and the slab. To choose the spike and slab variances, note that the two corresponding densities intersect at the points ±*σ* *ε* where $\varepsilon =\sqrt{(}2\log(c)c^{2}/(c^{2}-1))$. Then *δ* = *σ* *ε* can be interpreted as a threshold of practical significance in the sense that ‘all coefficients in the interval [−*δ*,+*δ*] can be interpreted as practically zero’ 18. So given *c*, *σ* can be selected to detect a ‘zero effect’ with required accuracy *δ*. Larger values of *c* induce higher slab variance, allowing large effects to take on arbitrarily large values and encouraging stronger penalisation of small non‐zero effects. Larger values of *c* are therefore more suited to sparse underlying models. On the other hand, small values of *c* reflect the belief that there are few zero effects and therefore are suited to non‐sparse models. For example, *δ* = 0.1 and *c* = 10 correspond to variances for spike and slab *σ* ^2^=0.0021 and *σ* ^2^ *c* ^2^=0.21, respectively, while for *δ* = 0.1 and *c* = 100, *σ* ^2^=0.001 and *σ* ^2^ *c* ^2^=10. Variable selection can be carried out by selecting variables with posterior inclusion probability (i.e. the mean of the posterior distribution of *γ*_*j*_) of more than 0.5. The shrinkage properties of SSVS are sensitive to the shape of the spike and the slab. Rockova *et al.*, 18, who focused on variable selection, considered three combinations of *c* and *δ*, two of which were suitable for aggressive shrinkage (*c* = 100, *δ* = 0.05 or 0.1) and one for ‘softer’ shrinkage (*c* = 10, *δ* = 0.1). In this work, which focuses on risk prediction, we choose the values of hyperparameter *c* for a given *δ* using cross‐validation. Specifically, unless stated otherwise, we select the value of *c* using cross‐validation over a grid of values (*c* = 3,5,8,10,15,20,30or50) for fixed *δ* = 0.1.

In one variant of SSVS, Ishwaran *et al.*, 37 suggested moving the mixture element one level down, that is, on the variances rather than on the regression coefficients directly. They placed inverse gamma priors on the slab and spike variances and called this approach ‘normal mixture of inverse gamma’ (NMIG). O'Hara and Sillanpaa 38 provide a nice review of the Bayesian approach to model selection. In this work, we applied the SSVS and NMIG approaches, which gave almost identical results, and so we only present results from SSVS.

#### 2.2.3 Credible intervals for regression coefficients and predicted probabilities

The estimated posterior distribution of the regression coefficients can be easily used to obtain credible intervals for regression coefficients and predicted probabilities. However, a note of caution should be added here regarding the frequentist coverage of Bayesian credible intervals. Bayesian credible intervals should have their corresponding *asymptotic* frequentist coverage provided that the prior distribution assigns a non‐zero probability to the true value of the quantities to be estimated. However, in the scenarios considered in this paper, the sample sizes are relatively small and the prior distributions are informative, and so the asymptotic properties may not apply. As a result, the correct frequentist coverage of Bayesian credible intervals is not guaranteed. This was noted by Kyung *et al.*, 16, who did not present any coverage results in their simulation studies, and more recently by Efron 17.

### 2.3 Software

All the analyses and simulations were carried out in R. We used the package ‘glmnet’ to fit models using ridge, lasso and adaptive lasso and the packages ‘pensim’ for elastic net and ‘ncvreg’ for SCAD. The tuning parameters were selected using 10‐fold cross‐validation. For Bayesian methods, we used JAGS to compile the models and obtained the samples using the R‐JAGS interface provided by the package ‘rjags’. We used 15 000 iterations with 5000 burn‐in sample. The Gelman–Rubin diagnostics were used to verify that parallel chains converged to the same posterior distribution. In the Supporting Information material (Section S1), we provide details and the functions used to fit the Bayesian lasso and SSVS using R and JAGS.

## 3 A case study: penile cancer

Penile cancer is a rare disease. Over an 18‐year period, 128 patients were diagnosed with invasive squamous cell carcinoma of the penis and treated within the North London Cancer Network. For this case study, the outcome of interest is defined as death within 5 years from the time of diagnosis. Twenty‐five deaths were observed, and we considered nine potential predictors. Five of them were continuous: three biomarkers, proteins that reflect aggressive cell cycle phenotypes 7 (‘Ki67’, ‘Mcm2’ and ‘Ki67‐g95’), age at diagnosis (‘age’) and depth of invasion (‘depthin’). The rest were binary: lymph node status (‘lymphnode’), lymphovascular invasion (‘vascinv’), tumour extent (‘extent’) and DNA ploidy status (‘ploidy’). A logistic regression model was fitted using the nine predictors and the EPV was just 2.8(=25/9). The following abbreviations are used for the methods: ENET, elastic net; ALASSO, adaptive lasso, BLASSO, Bayesian lasso; SSVS, stochastic search variable selection.

Here, we aim to illustrate the application of penalised regression methods for a binary outcome (death from penile cancer), and we focus on the shrinkage‐related properties of the methods rather than on their predictive performance, which is assessed in the next section. For the SSVS method, in addition to using cross‐validation to choose the value of *c*, we present the results obtained when *c* was fixed to equal 30. Because the value selected by cross‐validation was *c* = 10, the additional use of *c* = 30 allows us to illustrate how the results depend on the value of this parameter.

In Table 1, we present standardised coefficient estimates (i.e. after scaling the predictors to have mean zero and variance one) for each method and percentage shrinkage compared with MLE. The coefficients from MLE vary substantially in magnitude, from −0.20 (biomarker ‘Ki67’) to 1.34 (lymph node status). Overall, ridge tends to shrink the MLE estimates more uniformly (apart from the three biomarkers) than the other methods. Adaptive lasso, SCAD, Bayesian lasso and SSVS with *c* = 30 shrink the most coefficients to zero (5), followed by lasso and elastic net (3). Regarding the shrinkage properties of adaptive lasso and SCAD, it is observed that small coefficients tend to be shrunk to zero, whereas large coefficients are shrunk less than small coefficients. For example, the coefficient of lymph node status (which is the largest estimated coefficient using MLE) is only shrunk by 16*%* and 5*%* for adaptive lasso and SCAD, respectively, in comparison with 25*%* for lasso and 32*%* for ridge.

**Table 1:** Penile cancer case study: standardised regression coefficient estimates and percentage shrinkage (in parentheses) compared with MLE.

	MLE	RIDGE	LASSO	ENET	ALASSO	SCAD	BLASSO	SSVS (*c*=10)	SSVS (*c*=30)
age	1.03	0.60 (42)	0.56 (46)	0.58 (44)	0.58 (44)	0.31 (70)	0.75 (28)	0.60 (42)	0.89 (14)
depthin	0.85	0.54 (37)	0.60 (29)	0.59 (30)	0.69 (19)	0.44 (48)	0.67 (21)	0.58 (31)	0.88 (−4)
Ki67	−0.20	−0.06 (70)	0.00 (100)	0.00 (100)	0.00 (100)	0.00 (100)	0.00 (100)	−0.01 (93)	0.00 (100)
Mcm2	−0.58	−0.01 (98)	0.00 (100)	0.00 (100)	0.00 (100)	0.00 (100)	0.00 (100)	0.01 (98)	0.00 (100)
Ki67‐g95	0.70	0.17 (75)	0.00 (100)	0.00 (100)	0.00 (100)	0.00 (100)	0.00 (100)	0.12 (82)	0.00 (100)
lymphnode	1.34	0.91 (32)	1.01 (25)	1.00 (25)	1.12 (16)	1.27 (5)	1.17 (13)	0.95 (29)	1.32 (2)
vascinv	0.35	0.23 (34)	0.08 (79)	0.10 (72)	0.00 (100)	0.00 (100)	0.00 (100)	0.18 (51)	0.00 (100)
extent	0.36	0.24 (33)	0.13 (65)	0.15 (59)	0.00 (100)	0.01 (97)	0.00 (100)	0.19 (49)	0.00 (100)
ploidy	0.71	0.37 (49)	0.22 (69)	0.25 (65)	0.08 (89)	0.00 (100)	0.39 (45)	0.30 (57)	0.38 (46)

For SSVS, *c* = 10 was the value selected using cross‐validation. MLE, maximum likelihood estimation; BE, backwards elimination; LSF, linear shrinkage factor; ENET, elastic net; ALASSO, adaptive lasso; BLASSO, Bayesian lasso; SSVS, stochastic search variable selection; SCAD, smoothly clipped absolute deviation.

Bayesian lasso tends to shrink large non‐zero coefficients less than small ones due to the heavy tails of the double exponential prior distribution. So, in comparison with lasso, Bayesian lasso shrinks (hard shrinkage based on a threshold of one standard deviation from the posterior mean) more coefficients to zero (5) and shrinks the large coefficients less. For example, the effect of lymph node status is shrunk by 13*%* using Bayesian lasso compared with 25*%* using the classical lasso. SSVS with *c* = 10 features a uniform shrinkage pattern, similar to ridge, without shrinking any coefficients to zero. In contrast, SSVS with *c* = 30 applies much more aggressive shrinkage; it shrinks five coefficients to zero, whereas large coefficients are shrunk less than small coefficients, similar to adaptive lasso, SCAD and Bayesian lasso. For instance, the coefficient of lymph node status is shrunk by 29*%* when *c* = 10 but only by 2*%* when *c* = 30.

Having examined and contrasted the shrinkage properties of the methods through this case study, we shall, in Sections 4 and 5, compare their predictive performance in a variety of scenarios using simulation. The predictive performance of each method depends on the particular features of the data set in hand, such as strength of effects, presence of noise predictors and correlation between predictors.

## 4 Simulations based on the penile cancer data set

### 4.1 Simulation settings

Using the data from the penile cancer data set, we simulated data with varying EPV. Let *Y*_*i*_ and ***X***_*i*_ be the binary outcome and the vector of covariates, respectively, for the *i*th patient (*i* = 1,…,*N*), where *N* is the number of patients in the original data set. Suppressing the indicator for the patient, *i*, the assumed regression model is of the form logit(*E*(*Y*)) = ***X*** ^*T*^ ***β***. We simulate new data sets using the following steps:

1. Fit a logistic regression model using ridge regression (to avoid extreme values of regression coefficients) to the original data set to obtain $\hat{\beta }={\left({\hat{\beta }}_{0},{\hat{\beta }}_{1}^{T}\right)}^{T}$ where ${\hat{\beta }}_{0}$ corresponds to the estimate for the intercept term and ${\hat{\beta }}_{1}$ to the estimate of the vector of regression coefficients for the predictors.
2. Choose the prevalence (*prev*) and replace ${\hat{\beta }}_{0}$ by the value ${\hat{\beta }}_{0}^{*}$ that makes the average fitted probability equal to *prev*. Let $\beta^{*}={\left({\hat{\beta }}_{0}^{*},\hat{\beta_{1}^{T}}\right)}^{T}$ .
3. To create a validation data set, sample *M* × *N* (where *M* is large – we used *M* = 50) ***X***′*s* with replacement from the original data set. Simulate a *Y*_*val*_ value for each of the *M* × *N* ***X***'s under the true model: *Y*_*val*_∼Bernoulli(logit^−1^(***X*** ^*T*^ ***β*** ^∗^)). The *M* × *N* values of (***X***,*Y*_*val*_) make up the validation data set. Repeat this procedure to produce 500 validation data sets.
4. To create a training data set, choose the EPV and calculate the sample size that corresponds to the selected EPV given the number of predictors (*p*) and the prevalence: $n=\frac{\mathrm{EPV}\times p}{\mathrm{prev}}$. Sample with replacement *n* ***X***′*s* from the original data set. Generate a *Y*_*sim*_ value for each of the *n* values of ***X*** under the same true model: *Y*_*sim*_∼Bernoulli(logit^−1^(***X*** ^*T*^ ***β*** ^∗^)). The *n* values of (***X***,*Y*_*sim*_) make up the training data set. Repeat this procedure to produce 500 training data sets.
5. Fit the prediction model (using MLE and penalised methods) to each of the training data sets, apply the fitted model to the corresponding validation data set to compute the following performance measures: calibration slope, C‐statistic and predictive mean square error (Section 4.3).

### 4.2 Model‐fitting methods

We fit the models using MLE and a selection of frequentist (LSF, RIDGE, LASSO, ENET, ALASSO and SCAD) and Bayesian (BLASSO and SSVS) shrinkage methods. We also present the performance of MLE following a stepwise selection procedure using backwards elimination based on the AIC criterion 39. For SSVS, the value of *c* was chosen by cross‐validation and a coefficient was set to zero if the posterior inclusion probability was less than 0.5, as explained in Section 2.2.2. For the Bayesian lasso, the hyperprior used for *λ* ^2^ was a Gamma distribution with shape parameter, *a* = 1 and rate parameter, *b* = 0.01 (as used in 18), and the decision threshold for hard shrinkage was one standard deviation around the posterior mean.

### 4.3 Performance measures

We compare the predictive performance of the various methods in terms of calibration, discrimination and root predictive mean square error (RPMSE) in two low EPV scenarios (EPV 3 or 5). We use the calibration slope to measure calibration 40. To calculate the calibration slope, the binary outcome is regressed on the prognostic index. The estimated slope in this regression is the calibration slope. A slope of one suggests perfect calibration. A slope of <1 suggests overfitting; >1 suggests underfitting. We measure discrimination, the ability of the model to discriminate between high‐ and low‐risk patients, using the C‐statistic 41. If we consider two discordant patients, that is, one who has the event and one who does not, C‐statistic is the probability that the patient who experienced the event has a higher predicted probability. A value of 0.5 suggests that the model has no discriminatory ability, while a value of 1 suggests that the model can discriminate perfectly between higher‐risk and lower‐risk patients. Finally, the RPMSE is the square root of the average squared difference between the true and estimated predicted probabilities. The lower the RPMSE, the more accurate predictions the model provides. We present the results using vertical box‐plots, each box‐plot summarising a performance measure for each method. For each measure, the dotted red horizontal line is the median value when MLE is used. For the calibration slope, we also add a blue horizontal line that corresponds to perfect calibration (1). There was a small number of outliers for each method, and these are not shown for ease of presentation. Also, in a small number of simulated data sets (<2*%*) when *E* *P* *V* = 3 some of the methods, for example, lasso, shrank all coefficients to zero or very close to zero, and hence, the calibration slope could not be estimated. Such instances were removed for the presentation of results for all methods. For each scenario and each performance measure, we state the maximum Monte Carlo simulation error (for the median) among methods.

### 4.4 Coverage of credible and confidence intervals for predicted probabilities

We also investigate the coverage properties of credible and confidence intervals for the predicted probabilities. To better assess coverage, for this simulation, we generated 1000 training data sets with varying EPV (3, 5 and 10) as described in Section 4.1. Each patient's true risk is obtained using the (known) true values of the regression coefficients and the observed values of their predictor variables. For each patient, a 90*%* credible (or confidence) interval for his true risk is calculated in every simulation, and the coverage probability for that patient is the proportion of times his true risk is contained in that interval. We examine the coverage of these intervals for the true risks of the 128 patients in the original cohort using the Bayesian lasso, SSVS and frequentist ridge and lasso, and also MLE.

### 4.5 Bias of the estimates of regression coefficients

In low EPV scenarios, the risk of the event tends to be underestimated for lower‐risk patients and to be overestimated in higher‐risk patients when standard MLE is used to fit the model and model performance is assessed in new data. The main aim of using shrinkage methods is to decrease the prediction error by shrinking predictions towards the average risk, thus reducing the range of the predicted risks. However, this is achieved at the cost of introducing some bias towards the null in the coefficient estimates. Therefore, we present information about the bias in the coefficient estimates for each method, specifically the median difference between the true and estimated value of a coefficient (median bias) as a percentage of the true value of the coefficient.

### 4.6 Results

The prognostic strength of the risk model for the penile cancer data (128 patients, 25 events, 9 predictors) was relatively high, with the ‘true’ (standardised) coefficients ranging from −0.01 to 0.89 (true coefficients for predictors were as follows: age: 0.58; depth of invasion: 0.52; Ki67: −0.06; Mcm2: −0.01, Ki67‐g95: 0.17; lymph node status: 0.89; lymphovascular invasion: 0.23; extent of invasion:0.24; ploidy status: 0.36).

#### 4.6.1 Predictive performance

**Figure 1:**
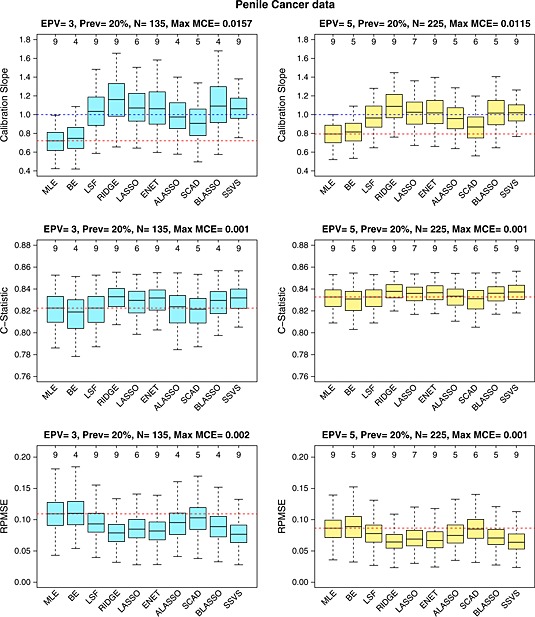
Performance measures for the penile cancer data: calibration, discrimination and predictive accuracy for *E* *P* *V* = 3 (left) or 5 (right). The number on top of each graph is the median number of predictors selected by each method. The red horizontal line is the median value for MLE. The blue horizontal line is the optimal calibration slope. EPV, events per variable; N, number of observations; RPMSE, root predictive mean squared error; MLE, maximum likelihood estimation; BE, backwards elimination; LSF, linear shrinkage factor; ENET, elastic net; ALASSO, adaptive lasso; BLASSO, Bayesian lasso; SSVS, stochastic search variable selection; MACE, Monte Carlo simulation error (for the median). The number of data sets (for each method) where the calibration slope could not be estimated for *E* *P* *V* = 3 was as follows: LSF: 2; Ridge: 1; Lasso: 1; ENET: 2; BLASSO: 3; Alasso: 1; SCAD: 2; SSVS: 0.

The predictive performance of the methods is shown in Figure 1. In terms of calibration, all shrinkage methods offer improvement compared with MLE, while backwards elimination showed overfitting, very similar to MLE. LSF, lasso, elastic net, adaptive lasso and SSVS demonstrate good calibration; ridge (and Bayesian lasso, when *E* *P* *V* = 3, to a lesser degree) appears to underfit the model; SCAD shows signs of overfitting especially for *E* *P* *V* = 5. The penalised methods tend to discriminate slightly better (except for SCAD and adaptive lasso) and to have lower RPMSE (except for SCAD when *E* *P* *V* = 5) than MLE, and this is more evident in the lowest EPV scenario. Overall ridge, elastic net and SSVS performed best. The least accurate shrinkage methods, adaptive lasso and SCAD, selected the fewest variables (with the exception of Bayesian lasso). SSVS gave results similar to elastic net, and the most commonly selected value for hyperparameter *c* when *E* *P* *V* = 3 was *c* = 10 (43% of the times), followed by *c* = 8 (37% of the times).

#### 4.6.2 Coverage of credible and confidence intervals for predicted probabilities

**Figure 2:**
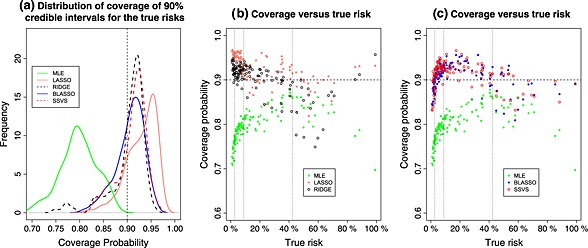
(a) Distribution of coverage probabilities of out of sample predictions (MLE, ridge, lasso, Bayesian lasso and SSVS) for 128 patients for the penile cancer data when *E* *P* *V* = 5. (b),(c): Coverage probability versus true risks. Dashed lines (for all plots) show the nominal coverage (90%). Dotted vertical lines for plots (b) and (c) show the 15th/50th/85th percentiles of the true risks. MLE, maximum likelihood estimation; SSVS, stochastic search variable selection.

The distribution of coverage probabilities for MLE, ridge, lasso, Bayesian Lasso and SSVS for *E* *P* *V* = 5 is shown in Figure 2(a). The coverage for MLE was poor, and use of shrinkage methods generally resulted in improved coverage. However, as shown in Figure 2(b), the coverage for lasso was above the nominal level for low‐risk patients, while for ridge, the coverage was slightly above the nominal level for low‐risk patients and below the nominal level for very high‐risk patients (e.g. >85th percentile). For Bayesian lasso and SSVS (Figure 2(c)), the coverage was slightly below the nominal level for patients with very low (e.g. <15th percentile) or very high (e.g. >85th percentile) risk and slightly above the nominal level for patients with risk in between these values. The coverage of all methods worsened as the EPV dropped and improved as the EPV increased (results not shown).

#### 4.6.3 Bias considerations

**Figure 3:**
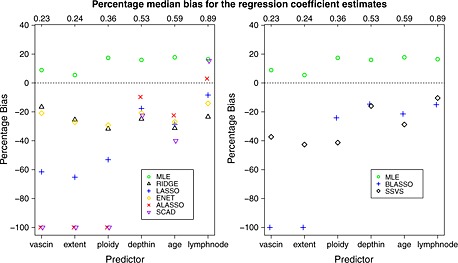
Percentage median bias of the estimates for each of the six coefficients for the penile cancer data with *E* *P* *V* = 3: frequentist (left) and Bayesian (right) shrinkage methods and MLE. The numbers on top of each graphs are the true values of the six regression coefficients. MLE, maximum likelihood estimation; ENET, elastic net; ALASSO, adaptive lasso; BLASSO, Bayesian lasso; SSVS, stochastic search variable selection; SCAD, smoothly clipped absolute deviation.

Figure 3 shows the percentage median bias in the coefficient estimates (calculated using 500 simulations) for each of the six largest regression coefficients. Ridge tends to shrink all coefficients by a similar factor. A similar pattern is observed for SSVS and elastic net in this example. The amount of bias introduced by lasso depends on the true effect sizes with larger relative bias for smaller effects. This differential bias is even more pronounced for adaptive Lasso, SCAD and Bayesian lasso, which shrink the smallest effects to zero for most simulated data sets, while the bias for the largest effects is relatively small. While unbiased estimation of coefficients is important when the aim is to investigate associations, bias is considered to be a less important issue for risk prediction studies where the predictive performance of the model is of main interest.

## 5 Further simulations

In this section, we perform additional simulations for scenarios with noise and correlated predictors to aid understanding of the findings from the simulations based on real data. We also explore the sensitivity of our simulation results to the selection of tuning parameters for the frequentist methods by using one single cross‐validation versus using repeated cross‐validations.

### 5.1 Noise and correlated predictors

We used artificial data sets where both predictors and outcomes were generated to match a hypothetical scenario. The advantage of this simulation setting over simulations based on real data is that we have full control over the features of the data and specifically the distribution of covariates, their strength and the correlations between them. We explore two main scenarios: (i) the presence of noise predictors and (ii) the presence of correlated predictors, both of which might affect the performance of the methods. The prevalence of the event for all simulation scenarios in this section was set to either 15*%* or 20*%*.

For (i), we consider two special cases. We first consider a sparse scenario and investigate how the performance of methods that perform variable selection compares with those that do not. Second, we consider a special case motivated by a situation where researchers have to consider a large number of potential predictors, some of which are known from the literature to be predictive of the outcome, but others may not. It is of interest to explore how the predictive performance of MLE deteriorates as EPV drops by adding noise predictors to the set of true predictors and how much of the lost predictive accuracy can be recovered by the use of penalised methods. For (ii), we explore whether those methods designed to handle correlated predictors (e.g. ridge and elastic net) have better predictive performance than the others (e.g. lasso, which tends to only keep some predictors from a set of correlated predictors) when predictors are correlated. We now present a summary of the findings for each scenario; detailed results can be found in the Supporting Information material (Section S3).

#### 5.1.1 Noise predictors – model sparsity

We generated seven independent and normally distributed covariates. A sparse design was induced by specifying a single true predictor and six noise predictors with ***β***_1_=(1.3,0,0,0,0,0,0). The full results are presented in Figure S1 in the Supporting Information. In terms of calibration, all methods offered improvement compared with MLE, which produced an overfitted model (median calibration slope was 0.70 for *E* *P* *V* = 3). Bayesian lasso, lasso and ridge (to a lesser degree) produced underfitted models when *E* *P* *V* = 3. When *E* *P* *V* = 5, LSF, adaptive lasso, SCAD and SSVS produced slightly overfitted models. Regarding discrimination and RPMSE, methods that perform variable selection offered a noticeable improvement compared with MLE, ridge and LSF. Backwards elimination was also better than MLE. The median C‐statistic was 0.768 for MLE and ridge when *E* *P* *V* = 3,0.781 for backwards elimination, 0.788 for elastic net and between 0.788 and 0.794 for other methods. The pattern was similar for RPMSE. For MLE, the RPMSE was 0.0909, compared with 0.0745 for ridge, while the rest of the methods (with the exception of LSF and backwards elimination) were substantially more accurate, with RPMSE ranging from 0.0524 (elastic net) to 0.0237 (SSVS) for *E* *P* *V* = 3. Among the methods that perform variable selection, elastic net selected the most variables and SSVS and BLASSO the fewest (median number of selected variables 3, 1 and 1, respectively, for *E* *P* *V* = 3). All methods that perform variable selection selected the true predictor in all simulations, while SSVS and Bayesian lasso had the lowest false inclusion rate (Table S3 in the Supporting Information). In general, more shrinkage was applied by SSVS compared with the simulation without noise predictors (in Section 4), and the most commonly selected value of *c* was 30. Overall, the methods that perform aggressive variable selection, that is, SCAD, adaptive lasso, Bayesian lasso and SSVS, performed best.

#### 5.1.2 Noise predictors – addition of noise predictors

Fifteen predictors were independently generated from the standard normal distribution. Five of them were ‘true’ predictors, and the vector of their true coefficients was ***β***_1_=(1,0.8,0.6,0.4,0.2). The rest were noise predictors. We firstly considered a model with the five true predictors only and a model with the 10 noise predictors added. The EPV for the model with five true predictors was 6; it dropped to 2 when 10 noise predictors were added. The full results are presented in Figure S2 in the Supporting Information material. blackAs expected, the amount of overfitting by MLE increased with the addition of noise predictors, while most shrinkage methods had improved calibration. The models derived by backwards elimination were overfitted for both before and after the addition of the 10 of noise predictors. LSF, adaptive lasso and especially SCAD produced an overfitted model for the simulation with true predictors only. When the 10 noise predictors were added, ridge, lasso and Bayesian lasso produced underfitted models. All shrinkage methods had lower RPMSE than MLE. The improvements in discrimination were relatively small: for 10 noise predictors, the median C‐statistic was 0.767 for MLE, while among the penalised methods, adaptive lasso achieved the highest C‐statistic (0.780). blackIn general, shrinkage methods that perform variable selection had slightly better performance to ridge and LSF but with fewer number of predictors retained (median number of predictors was 10 for elastic net, 6 for SCAD, adaptive lasso and for Bayesian lasso, and in between for the other methods). blackFor SSVS, the most commonly selected value for *c* when there were no noise predictors was 15. After the addition of noise predictors, more shrinkage was applied by SSVS, and the most common choice of *c* was again 30.

#### 5.1.3 Correlated predictors

Seven continuous predictors were generated from a zero‐mean multivariate normal distribution. The first four predictors were highly correlated (pairwise correlation = 0.8), while the rest were independent of each other and of the first four predictors. The vector of true coefficients was ***β***_1_=(0.8,0.6,0.4,0.4,0.2,0.2,−0.2). All methods offered improvement compared with MLE (the full results are presented in Figure S3 in the Supporting Information material). Apart from SCAD, which slightly overfitted the model (with a median calibration slope of 0.89 for *E* *P* *V* = 3, compared with 0.81 of MLE), all shrinkage methods demonstrated good calibration, with signs of underfitting for ridge, lasso, elastic net and Bayesian lasso. For SSVS, the most commonly chosen value of *c* was 8. The predictive performance of lasso, Bayesian lasso, adaptive lasso and SCAD was generally worse than that of ridge and SSVS in terms of discrimination and RPMSE. The high correlation between predictors can cause these methods (lasso, Bayesian lasso, adaptive lasso and SCAD) to select at random a subset of the four correlated predictors, and this is likely to be at least part of the reason for the worse predictive accuracy of these methods. The median C‐statistic was 0.862 for MLE, 0.868 for lasso and 0.872 for ridge, when *E* *P* *V* = 3. Similarly, the RPMSE was 0.094 for MLE, 0.075 for lasso and 0.065 for ridge. The performance of elastic net and SSVS was very close to that of ridge, while SCAD and adaptive lasso were worse than lasso. We highlight the fact that when *E* *P* *V* = 3, lasso selected all four correlated predictors in only 30*%* of the simulated data sets, in comparison with 64*%* for elastic net which tended to select the correlated predictors as a group. When the pairwise correlation was changed to 0, the corresponding proportion was 68*%* for lasso and its predictive performance became very close to that of ridge (results not shown).

### 5.2 Selection of the tuning parameter

The selection of the tuning parameters for ridge, lasso, elastic net, adaptive lasso and SCAD is most often made using *k*‐fold cross‐validation. This makes the selection of the tuning parameters non‐deterministic, that is, for a different fold assignment to the observations, a different tuning parameter may be selected (unless ‘leave‐one‐out’ cross‐validation, i.e. *k* = *n*, is used). In principle, using only a single *k*‐fold cross‐validation may affect the stability of the results in that a different *k*‐fold split might yield a different value for the tuning parameter. One way to overcome this issue is to perform *k*‐fold cross‐validations many times, say 50, for a given data set, thus obtaining 50 values for the tuning parameter, and then use the value that corresponds to a particular percentile, *θ*, of those 50 values. We term this approach ‘repeated cross‐validations’. Roberts and Nowak 42 studied the instability of lasso in terms of variable selection and suggested that choosing a large value of *θ* (they argue that *θ* = 0.95 is appropriate in most scenarios) considerably reduces the variable selection instability and model selection error. They also found that it may improve predictive performance by protecting against extremely large prediction errors.

In the simulation studies reported in previous sections, we used 10‐fold cross‐validation (non‐repeated), which is the default option in most packages, and in this section, we further investigate the effect of using a single cross‐validation in a sub‐study for the penile cancer example of Section 4 and the sparse scenario of Section 5. In particular, we compared the results when using a single cross‐validation versus repeated cross‐validation. We considered two possible percentiles: *θ* = 0.5 and *θ* = 0.95. The results for the single cross‐validation method and the repeated cross‐validation method with *θ* = 0.5 were very similar (Figures S4 and S5 in the Supporting Information material), suggesting that use of single cross‐validation did not affect our conclusions. However, use of the repeated cross‐validation method with *θ* = 0.95 resulted in underfitted models. This is because the use of *θ* = 0.95 tends to select a larger value for the shrinkage parameter than does the use of *θ* = 0.5. This causes more aggressive shrinkage, which then leads to more underfitting. When analysing a real data set, we suggest using repeated cross‐validations with *θ* = 0.5, as we do in the next section.

## 6 Data illustration

Here, we demonstrate the application of shrinkage methods to a real data example with a low‐outcome prevalence. The aim is to derive a risk model for estimating the probability of thromboembolism in patients with hypertrophic cardiomyopathy. The event of interest was a thromboembolic event within 5 years of first evaluation. Predictors included age, sex, atrial fibrillation at baseline (‘af’), NYHA class of disease severity (three categories), history of prior thromboembolic events (‘stroke history’) or history of diabetes, hypertension or vascular disease (all binary) and also left atrial diameter (‘la diameter’), peak left ventricular outflow tract gradient (‘peak lvot’), maximum left ventricular wall thickness(‘mwt’) and its square term, fractional shortening(‘fs’) and left ventricular ejection fraction (‘lvef’) (all continuous).

We used the various methods to develop logistic regression prediction models from data on 2082 patients (75 events). There were 15 regression coefficients and so the EPV was only 5. The models were externally validated using data from different centres (2739 patients, 97 events). When using a frequentist shrinkage method, we selected the tuning parameter using repeated cross‐validations (as described in Section 5.2) with *θ* = 0.5. We compared the calibration and discrimination of the fitted models using the calibration slope and C‐statistic, as described earlier. For overall predictive accuracy, we used the Brier score defined as the average squared difference between the estimated probability and the observed outcome.

**Figure 4:**
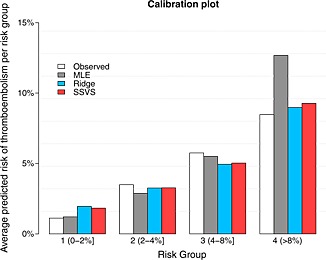
Calibration plot for the thromboembolism data. Number of patients are 983, 946, 574 and 236 for risk groups 1 to 4, respectively. MLE, maximum likelihood estimation; SSVS, stochastic search variable selection.

As in our simulation studies, backwards elimination, adaptive lasso, SCAD and Bayesian lasso selected the fewest predictors (eight), while lasso and SSVS(with *c* = 5 chosen using cross‐validation, for *δ* = 0.1) selected 12 and 13 predictors, respectively (Table 2). Elastic net retained all 15 predictors. All methods improved calibration (in the validation data) compared with MLE; ridge, lasso, elastic net, Bayesian lasso and SSVS underfitted the model, but this did not affect the other performance measures (Table 3). With the exception of SCAD, all shrinkage methods improved discrimination slightly, with ridge achieving the highest C‐statistic of 0.724, in comparison with 0.704 for MLE. All shrinkage methods had lower Brier scores than MLE, with ridge, lasso, elastic net and SSVS achieving the lowest. We also calculated the performance measures for the models produced from each of the 50 10‐fold cross‐validations for each method, to quantify the variability resulting from the selection of tuning parameters via non‐repeated cross‐validation. Some variability was observed in the calibration slope, C‐statistic and Brier score. For ridge regression, for example, the interquartile range across the 50 non‐repeated 10‐fold cross‐validations was [1.24, 1.35] for the calibration slope and [0.724, 0.726] for the C‐statistic. The variability was similar for the other methods. To visually assess the calibration of the model, we used the ‘calibration plot’ in Figure 4. It shows (for the validation data set) the observed proportion of patients with the event and the average predicted risks in four clinically meaningful groups of patients (defined by the predicted risks from MLE, which are more widely dispersed that the predicted risks of the other methods). MLE overestimates the risk of thromboembolism for the highest risk‐group, while ridge and SSVS, which were among the best performing methods, satisfactorily estimate the average risk in each of the four risk groups.

**Table 2:** Modelling the probability of a thromboembolic event.

	MLE	BE	RIDGE	LASSO	ENET	ALASSO	SCAD	BLASSO	SSVS
Intercept	−3.29	−3.27	−3.26	−3.26	−3.27	−3.36	−3.31	−3.23	−3.25
age	0.26	0.27	0.15	0.13	0.17	0.05	0.10	0.15	0.15
la diameter	0.36	0.35	0.24	0.30	0.26	0.43	0.39	0.25	0.31
mwt	0.49	0.47	0.19	0.21	0.23	0.07	0.21	0.21	0.25
mwt^2^	−0.34	−0.32	−0.15	−0.16	−0.18	−0.07	−0.15	−0.18	−0.21
peak lvot	−0.10	0.00	−0.03	0.00	−0.04	0.00	0.00	0.00	0.00
fs	−0.13	0.00	−0.05	0.00	−0.07	0.00	0.00	0.00	−0.05
lvef	−0.16	0.00	−0.07	−0.01	−0.08	0.00	0.00	0.00	−0.08
af	0.14	0.16	0.13	0.11	0.14	0.07	0.12	0.13	0.10
stroke history	0.22	0.22	0.17	0.17	0.18	0.19	0.20	0.17	0.16
female	−0.18	−0.20	−0.10	−0.09	−0.11	−0.08	−0.11	−0.10	−0.10
NYHA class II	0.13	0.00	0.09	0.02	0.10	0.00	0.00	0.00	0.07
NYHA class III/IV	0.10	0.00	0.08	0.01	0.09	0.00	0.00	0.00	0.05
vascular disease	−0.07	0.00	−0.03	0.00	−0.03	0.00	0.00	0.00	0.00
hypertension	−0.20	−0.22	−0.10	−0.06	−0.12	−0.01	−0.05	−0.11	−0.11
diabetes	−0.12	0.00	−0.06	−0.01	−0.07	0.00	0.00	0.00	−0.07
Number of
predictors retained	15	8	15	12	15	8	8	8	13

Standardised coefficients of logistic regression model estimated by MLE, BE and the shrinkage methods using a sample of 2082 patients and 75 events (*E* *P* *V* = 5). MLE, maximum likelihood estimation; BE, backwards elimination; LSF, linear shrinkage factor; ENET, elastic net; ALASSO, adaptive lasso; BLASSO, Bayesian lasso; SSVS, stochastic search variable selection; SCAD, smoothly clipped absolute deviation.

**Table 3:** External validation of the models for the probability of a thromboembolic event.

Performance measure	Calibration slope (s.e.)	C‐statistic (s.e.)	Brier score
MLE	0.78 (0.12)	0.704 (0.025)	0.03396
BE	0.85 (0.13)	0.703 (0.025)	0.03381
RIDGE	1.27 (0.18)	0.724 (0.024)	0.03357
LASSO	1.23 (0.17)	0.718 (0.024)	0.03359
ENET	1.22 (0.17)	0.723 (0.024)	0.03358
ALASSO	1.07 (0.16)	0.715 (0.026)	0.03361
SCAD	1.07 (0.16)	0.697 (0.026)	0.03370
BLASSO	1.26 (0.17)	0.723 (0.024)	0.03360
SSVS	1.19 (0.17)	0.718 (0.024)	0.03359

Performance measures were evaluated on a validation data set with 2739 patients and 97 events. MLE, maximum likelihood estimation; BE, backwards elimination; LSF, linear shrinkage factor; ENET, elastic net; ALASSO, adaptive lasso; BLASSO, Bayesian lasso; SSVS, stochastic search variable selection; SCAD, smoothly clipped absolute deviation.

## 7 Discussion

The application of penalised methods in low‐dimensional settings, although important, has been limited. In this paper, we investigated the use of penalised methods to alleviate the problem of model overfitting in low‐dimensional data with few events in the context of risk prediction. In particular, we focused on scenarios with EPV less than 10, where the danger of overfitting is particularly pronounced.

We examined three main categories of shrinkage methods. The first category includes methods that shrink the coefficients but do not perform variable selection (LSF and ridge). In our simulation studies, ridge produced slightly underfitted models in some cases but generally performed better than LSF in terms of discrimination and predictive mean square error. The second category includes frequentist penalised methods that also perform variable selection (lasso, elastic net, adaptive lasso and SCAD). Lasso and elastic net performed well in most scenarios. Elastic net was found to select more variables than lasso and was superior to lasso in scenarios with correlated predictors. Adaptive lasso and SCAD selected fewer variables than lasso and were better than lasso only in scenarios with many noise predictors. One motivation for considering the third category, Bayesian approaches, is to apply shrinkage and perform variable selection while also obtaining measures of uncertainty for the coefficients and predicted probabilities. When the objective is to obtain a simple model with few predictors, assessment of the uncertainty around variable selection decisions can be based on the posterior distribution of the coefficients (or the posterior inclusion probabilities). We considered Bayesian lasso and SSVS. Bayesian lasso was best suited to scenarios with noise predictors, whereas SSVS performed well in all scenarios.

Table 4 summarises our recommendations regarding the choice of method(s) in each of the scenarios considered. For each scenario, we highlight the methods that had the best predictive performance (denoted by ✓✓), the methods that performed better than MLE (denoted by ✓) and methods that should be avoided (denoted by ×).

**Table 4:** Recommended methods according to their predictive performance in data sets with few events.

	Scenario			
		Noise predictors		
Method	No noise predictors	Non‐sparse	Sparse	Correlated predictors
LSF	✓	✓	×	×
BE	×	×	×	×
RIDGE	✓✓	✓	×	✓✓
LASSO	×	✓✓	✓	✓
ENET	✓✓	✓✓	✓	✓✓
ALASSO	×	${✓}^{a}$	✓✓	×
SCAD	×	${✓}^{a}$	✓✓	×
BLASSO^b^	✓	✓✓	✓	✓
SSVS^c^	✓✓	✓✓	✓✓	✓✓

✓✓ Performed best in simulations.

✓ Performed well but was not the best method in simulations.

× Not recommended for the particular scenario.

a Tends to overfit the model in the presence of weak effects.

b Sensitive to the threshold selection for hard shrinkage.

c When the spike and slab variances are chosen appropriately using cross‐validation.

MLE, maximum likelihood estimation; BE, backwards elimination; LSF, linear shrinkage factor; ENET, elastic net; ALASSO, adaptive lasso; BLASSO, Bayesian lasso; SSVS, stochastic search variable selection; SCAD, smoothly clipped absolute deviation.

### 7.1 High‐dimensional settings

Penalised regression methods are widely used in high‐dimensional settings, often with highly sparse underlying models. For example, in genetic association studies, very few genetic markers are expected to be associated with the phenotype of interest. In such cases, sparse regression techniques such as lasso, adaptive lasso and SCAD can identify a small subset of relevant predictors and can provide good predictive accuracy. Breheny and Huang 27 compared SCAD with lasso in a gene expression and a genetic association study and noted that SCAD allows coefficients to take on large values much more easily than lasso. As a result, SCAD outperforms lasso when strong predictors (i.e. predictors with large coefficients) are present in the underlying true model. They also found that shrinkage applied by lasso is beneficial when predictors are weak; in such cases, SCAD tends to overfit the noisy data. Huang *et al* 43 explored the use of adaptive lasso in microarray data and linear regression. They found that adaptive lasso had lower predictive mean square error than lasso, except in cases where true predictors were correlated with the rest. Benner *et al.*, 26 used simulated microarray data and found that SCAD and adaptive lasso had the best predictive accuracy in highly sparse scenarios, while in moderately sparse scenarios, elastic net and lasso were better. In sparse scenarios, ridge performed poorly, whereas it performed well in one non‐sparse low‐dimensional setting considered. Austin *et al.*, 44 explored penalised methods and risk prediction in genome‐wide association studies. They found that SCAD and lasso were best for sparse models, while elastic net and ridge were suitable for non‐sparse models, which is in agreement with our results.

### 7.2 Alternative approaches

We have compared a wide range of methods, but alternative approaches also exist. For example, Firth's bias correction method 45 uses a different type of penalisation and deals well with problems involving separation 46. In the scenarios we studied, it showed some improvement compared with MLE in terms of prediction, but it was inferior to the other shrinkage methods considered, showing signs of overfitting (results not shown). In the Bayesian framework, alternative methodologies include the ‘model space’ approach, where the space of all possible models is considered and candidate models are suggested by the posterior model probabilities. Fast and efficient exploration of model and parameter space can be implemented using special samplers such as reversible jump MCMC. Parameter estimation and/or prediction can proceed by averaging over all or highly probable models. Also, techniques from machine learning, such as random forests, neural networks and support vector machines, have been used recently for prediction with medical data and have the potential to outperform standard regression methods in some cases, although recent studies showed that those methods require large EPV to fully realise their potential 47, and they may overfit if not carefully applied. Also, the results obtained from some of these non‐regression‐based approaches, for example, neural networks and support vector machines, may not be as intuitive and interpretable, posing concerns regarding reproducibility and transparency of the results 48.

### 7.3 Conclusion

In conclusion, it is important to consider penalised methods when developing prediction models for low‐dimensional data with few events. They can improve calibration and predictive accuracy compared with MLE, although improvement in discrimination was modest in the scenarios considered. One reason for this small improvement in discrimination is that, as penalised methods tend to shrink the predicted probabilities towards the average compared with MLE, the ordering of the predicted probabilities for patients with and without the event tends to remain unchanged (after shrinkage) for most patient pairs. Further research could consider alternative approaches to increase discrimination via better usage of patients with the event (e.g. super‐sampling patients with the event 49).

Backwards elimination, which is often employed by analysts to reduce the number of predictors and ostensibly meet the EPV recommendations, should be avoided in data sets with few events. We recommend using ridge regression or SSVS when no variable selection is required. When variable selection is required and no high correlations are observed between predictors, we suggest using lasso, while if there are high correlations, elastic net is the preferred option (this tends to select more variables than lasso). Adaptive lasso, SCAD and Bayesian lasso are better suited to scenarios with many noise predictors. In other cases, they can still be useful in identifying a set of strong predictors, but this set will tend to have reduced predictive accuracy compared with the models obtained by ridge and lasso. If a concise model comprising the strongest predictors is required and the analyst is prepared to sacrifice some predictive accuracy, then adaptive lasso, SCAD and also Bayesian lasso could be considered. SSVS can be applied in scenarios with and without noise predictors but fitting will be considerably slower than for the frequentist methods, especially if the hyperparameters are selected using cross‐validation. In general, Bayesian approaches are worth considering alongside the frequentist approaches due to their appealing features: variable selection based on posterior distributions; availability of credible intervals for predicted probabilities; ability to incorporate external information (e.g. from meta‐analysis) if available; and straightforward extension to the clustered‐data setting (all of which are topics for further research). Confidence/credible intervals for predicted probabilities from shrinkage methods had substantially better coverage than MLE, although there was evidence of under‐coverage or over‐coverage for some patients. Overall, no method outperformed the others in all scenarios. The choice of method should be made based on the features of the particular data set in hand. In addition, if one requires a simpler model, then a method that allows variable selection might be preferred.

## Supporting information

Supporting info item
