# Supplementary material for: Review and evaluation of penalised regression methods for risk prediction in low‐dimensional data with few events
Source: Stat Med. 2015 Oct 29;35(7):1159–77. doi: 10.1002/sim.6782 (PMC4982098; doi:10.1002/sim.6782)
Supplement: Supplementary file 1 — Supporting info item [file SIM-35-1159-s001.zip › artificial_correlated.pdf]

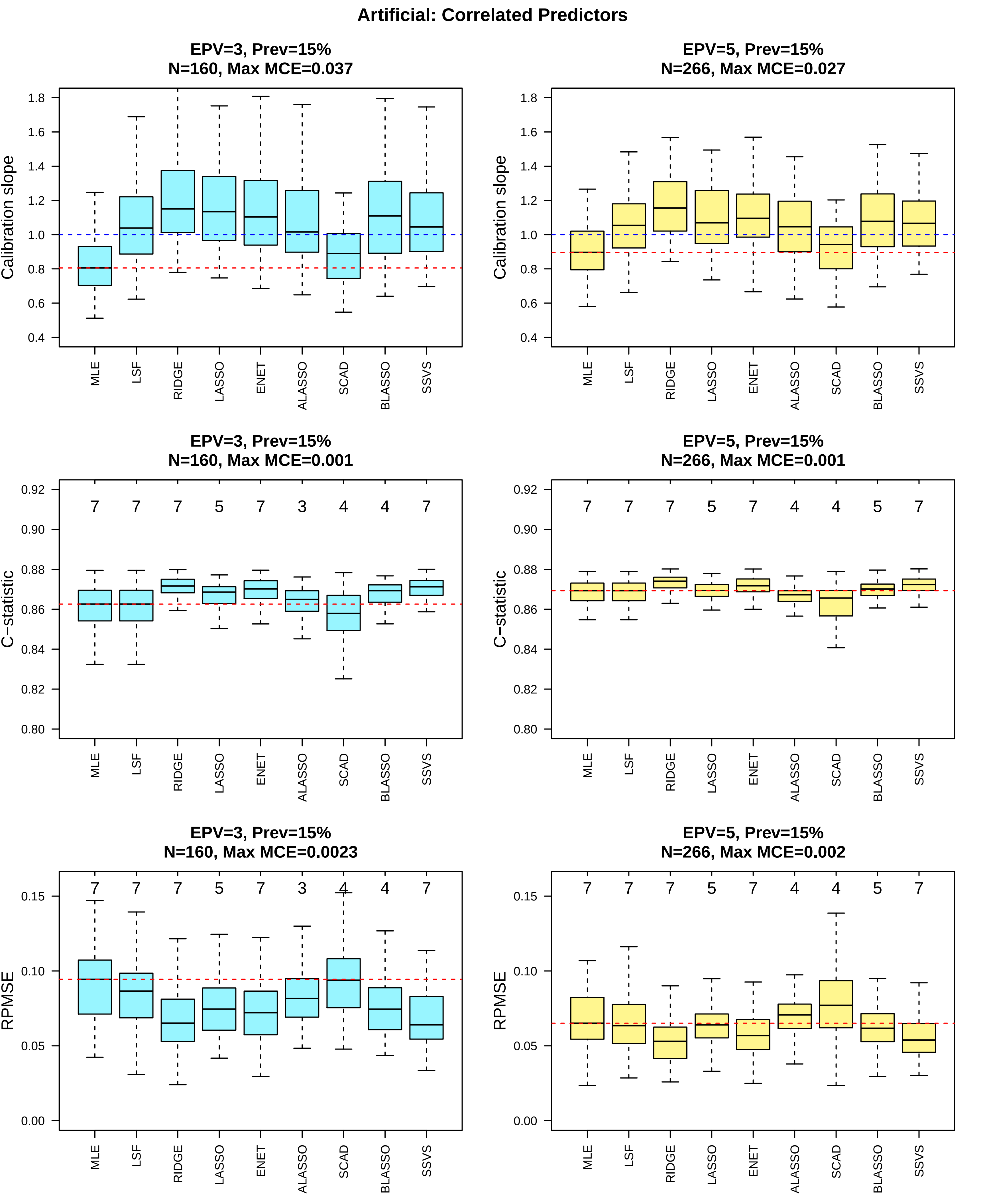
Artificial: Correlated Predictors
EPV=3, Prev=15%
N=160, Max MCE=0.037
EPV=5, Prev=15%
N=266, Max MCE=0.027
Calibration slope
EPV=3, Prev=15%
N=160, Max MCE=0.001
EPV=5, Prev=15%
N=266, Max MCE=0.001
C−statistic
7 7 7 5 7 3 4 4 7
7 7 7 5 7 4 4 5 7
EPV=3, Prev=15%
N=160, Max MCE=0.0023
EPV=5, Prev=15%
N=266, Max MCE=0.002
RPMSE
MLE
LSF
RIDGE
LASSO
ENET
ALASSO
SCAD
BLASSO
SSVS
