# Supplementary material for: Review and evaluation of penalised regression methods for risk prediction in low‐dimensional data with few events
Source: Stat Med. 2015 Oct 29;35(7):1159–77. doi: 10.1002/sim.6782 (PMC4982098; doi:10.1002/sim.6782)

Artificial: Sparse

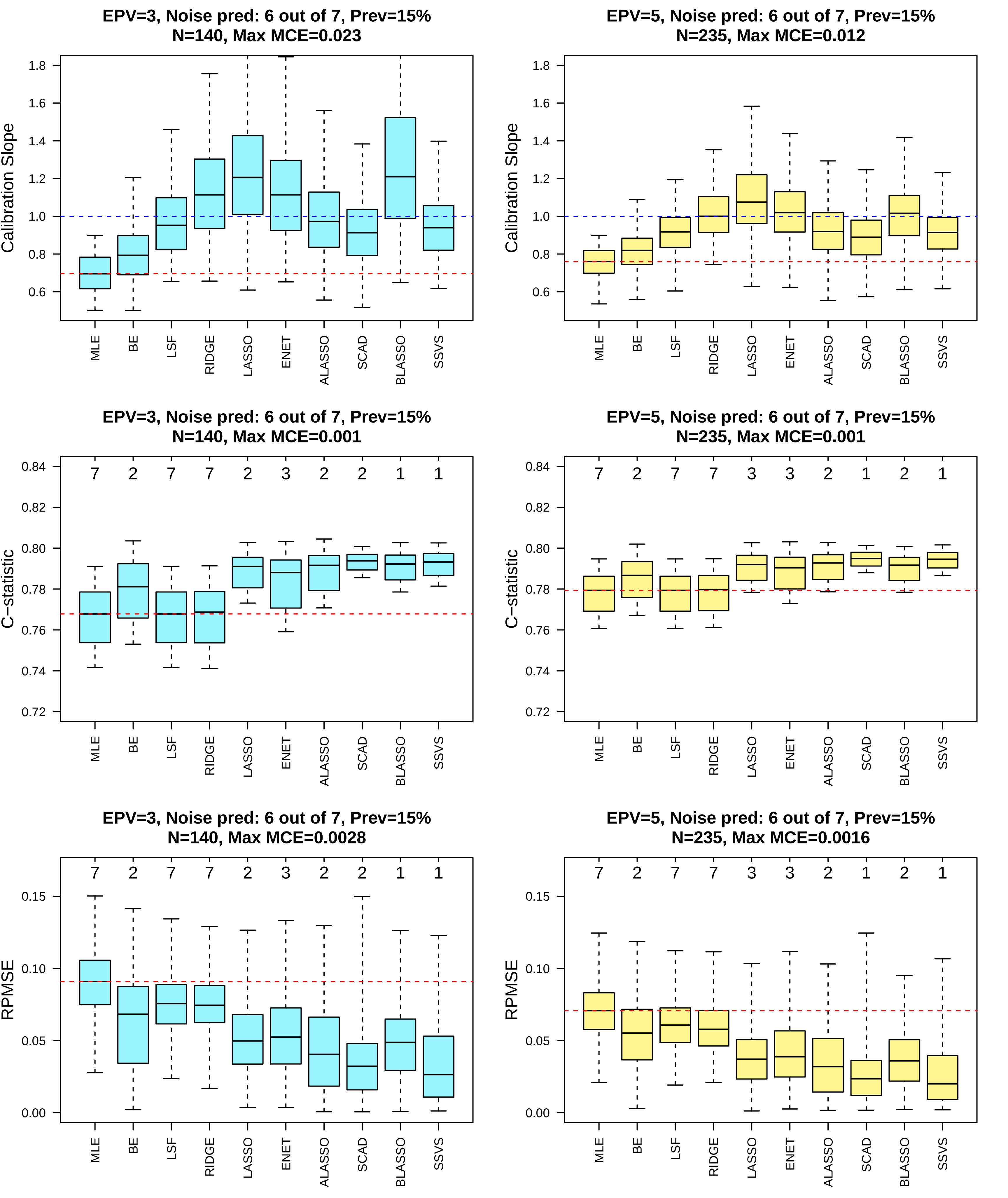

Supplement: Supplementary file 1 — Supporting info item [file SIM-35-1159-s001.zip › artificial_sparse.pdf]
