# Supplementary material for: Review and evaluation of penalised regression methods for risk prediction in low‐dimensional data with few events
Source: Stat Med. 2015 Oct 29;35(7):1159–77. doi: 10.1002/sim.6782 (PMC4982098; doi:10.1002/sim.6782)
Supplement: Supplementary file 1 — Supporting info item [file SIM-35-1159-s001.zip › artificial_sparse_repeated_cv.pdf]

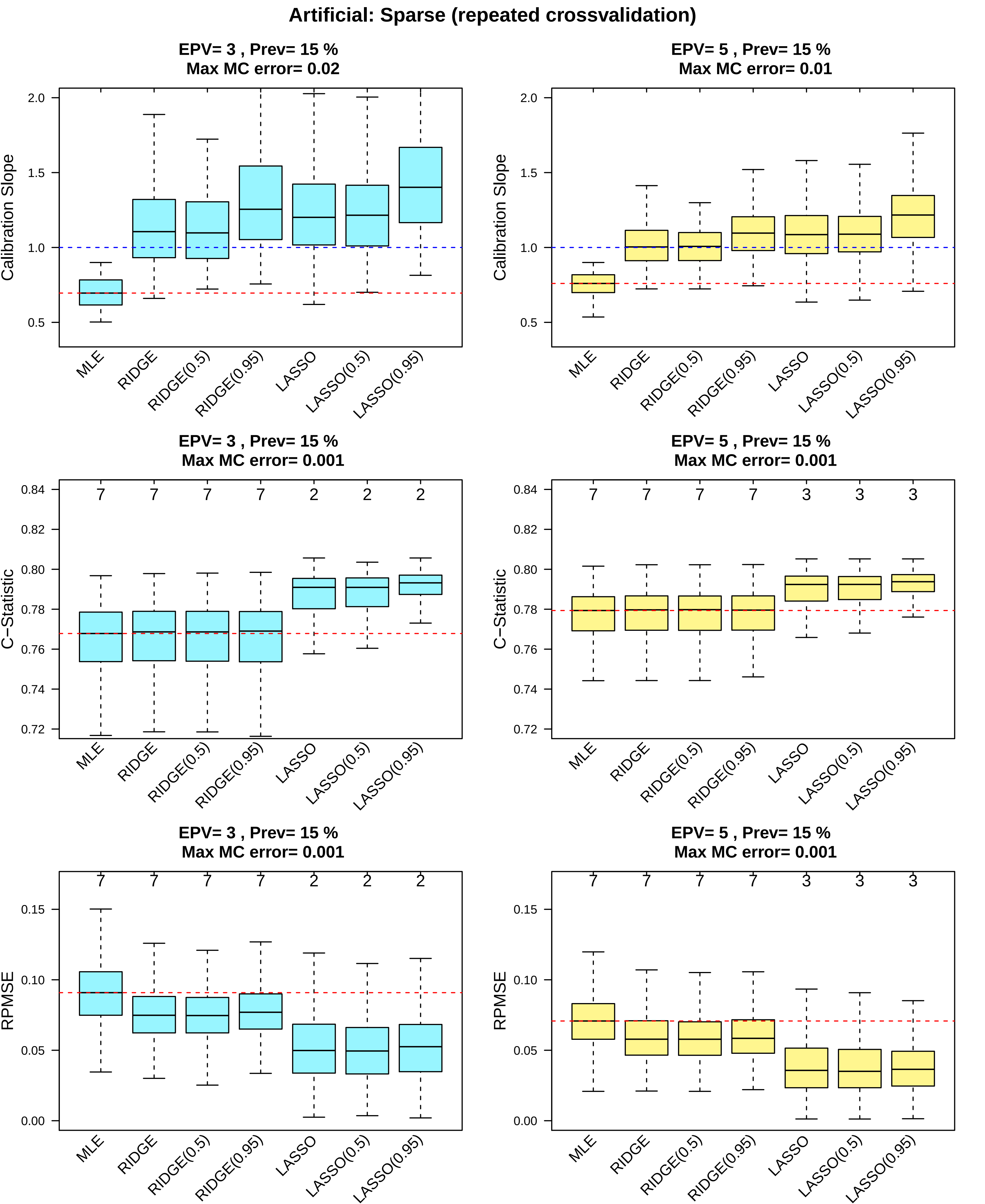
Artificial: Sparse (repeated crossvalidation)
EPV= 3 , Prev= 15 %
Max MC error= 0.02
EPV= 5 , Prev= 15 %
Max MC error= 0.01
Calibration Slope
EPV= 3 , Prev= 15 %
Max MC error= 0.001
EPV= 5 , Prev= 15 %
Max MC error= 0.001
C−Statistic
EPV= 3 , Prev= 15 %
Max MC error= 0.001
EPV= 5 , Prev= 15 %
Max MC error= 0.001
RPMSE
MLE
RIDGE
RIDGE(0.5)
RIDGE(0.95)
LASSO
LASSO(0.5)
LASSO(0.95)
