# Supplementary material for: Review and evaluation of penalised regression methods for risk prediction in low‐dimensional data with few events
Source: Stat Med. 2015 Oct 29;35(7):1159–77. doi: 10.1002/sim.6782 (PMC4982098; doi:10.1002/sim.6782)
Supplement: Supplementary file 1 — Supporting info item [file SIM-35-1159-s001.zip › Statmed_revision_2_sup.pdf]

Research Article

Statistics in Medicine

Received XXXX

(www.interscience.wiley.com) DOI: 10.1002/sim.0000

Supplementary material for the paper 'Review and Evaluation of Penalised Regression Methods for Risk Prediction in low-dimensional Data with Few Events'

Menelaos Pavlou [a] *, Gareth Ambler [a], Shaun Seaman [b], Maria De Iorio [a] and Rumana Omar [a]

S1. Demonstration of R and JAGS code

In this section we show how the Bayesian lasso logistic regression and Stochastic Search Variable Selection can be applied using JAGS and R. For the following it is necessary to have R (version 2.15 or later) and JAGS (version 3.3 or later) installed. The package 'rjacks' is required for the R to JAGS interface; packages 'mcmc' and 'coda' for obtaining samples from the posterior distributions.

We start with the Bayesian approaches. Firstly, the models are defined and saved in a file with 'jag' extension (this file will be complied in JAGS).

Bayesian lasso - JAGS model

```
model{
  for(i in 1:n){
  eta[i] <- beta0+inprod(x[i,],beta1[])
  pi[i] <- 1/(1+exp(-eta[i]))
  y[i] ~ dbern(pi[i])
  }
beta0~dnorm(0,0.001)
for (j in 1:q) {
   beta1[j] ~ dnorm(0,tausq)
```

John Wiley & Sons, Ltd, The Atrium, Southern Gate, Chichester, West Sussex, PO19 8SQ, UK.
[a] *Department of Statistical Science, University College London, London, WC1E 6BT, UK.*
[b] *MRC Biostatistics Unit, Cambridge, CB2 0SR, UK.*
* *Correspondence to: Department of Statistical Science, University College London, London, WC1E 6BT, UK. Email: m.pavlou@ucl.ac.uk*

Copyright © 0000 John Wiley & Sons, Ltd.

Prepared using simauth.cls *[Version: 2010/03/10 v3.00]*

```
  }
tausq<-1/sigmasq
sigmasq~dexp(0.5*lambdasq)
lambda<-sqrt(lambdasq)
lambdasq~dgamma(a,b)
}
```

Stochastic Search Variable Selection - JAGS model

```
model{
  for(i in 1:n){
  eta[i] <- beta0+inprod(x[i,],beta1[])
  pi[i] <- 1/(1+exp(-eta[i]))
  y[i] ~ dbern(pi[i])
 }
beta0~dnorm(0,0.01)
for (j in 1:q){
beta1[j] ~ dnorm(0,tausq[j])
tausq[j]<-(1-gamma[j])*(1/varspike)+gamma[j]*(1/varslab)
gamma[j]~dbern(p)
}
p~dunif(0,1)
}
```

The first and second files above are saved as ‘blasso.jag’ and ‘ssvs.jag’, respectively. Subsequently, the data and initial values for the parameters are provided to JAGS through R. Posterior samples and summaries from the MCMC samples are obtained using R. Below we present two functions which carry out these tasks. Note that in the second function we demonstrate how ‘parallel’ processing can be implemented to run each chain on a different core, for more time efficient sampling.

An R function to fit Bayesian lasso logistic regression

```
bayesian.lasso<-function(y,x,betain,a,b, n.iter,n.chains,burn.in){

#y=the vector of responses
#x= the model matrix
#a,b=hyperparmeters for the Gamma hyperprior
#betain= vector of initial values for the regression parameters
#n.iter=number of msmc samples
#n.chains=number of mcmc chains
#burn.in=number of samples to be discarded

#q=number of regression parameters
#n=sample size
q=length(betain)
n=length(y)
```

 Copyright © 0000 John Wiley & Sons, Ltd.

```
#Input the data as a list with named objects
data <- list("y" = y, "x" = x, "n" = n, "q"=q, "a"=a, "b"=b)

#Input initial values for the parameters a list with named objects
inits <- list(beta0=betain[1],beta1=betain[2:(q+1)])

#Use jags to fit the model, indicating to the directory of the relevant .jag file
jags.m <- jags.model(file="C:\\yourdirectory\\blasso.jag", data = data,
                    inits = inits, n.chains = n.chains, n.adapt = n.adapt)

#Define the quantities for which posterior distributions are required
params <- c("beta0","beta1","lambda")

#Use packages 'coda' and 'mcmc' to obtain the mcmc samples from the
#posterior distributions. First burn.in samples are discarded
samps <- coda.samples(jags.m, params, n.iter=n.iter)
samps<-mcmc(samps,start=burn.in+1)
samps<-mcmc.list(samps)
parameter.mean <- summary(samps)[[1]][,1]

#Obtain the mean of the posterior distribution for the regression and
#tuning parameters
beta=parameter.mean[1:(q+1)]
lambda=parameter.mean[q+2]

#Return estimates of the posterior means
return(list("beta"=beta,"lambda"=lambda))
}
```

An R function to fit SSVS logistic regression with parallel processing

```
bayesian.ssvspar<-function(y,x,betain,c,delta,n.iter,burn.in,n.chains){

#y=the vector of responses
#x= the model matrix
#betain= vector of initial values for the regression parameters
#delta= threshold of practical significance (see Rockova et al. (2013))
#c= the degree of separation between the spike and slab
#n.iter=number of msmc samples
#n.chains=number of mcmc chains
#burn.in=number of samples to be discared

#Calcalate the variance of lab and spike given c and delta
epsilon=sqrt( 2*log(c)*c^2/(c^2-1) )
varspike=(delta/epsilon)^2
```


Copyright © 0000 John Wiley & Sons, Ltd.

```
varslab=varspike*c^2

#Input the data as a list with named objects
data <- list("y" = y, "x" = x, "n" = n, "q"=q,"varslab"=varslab,"varspike"=varspike)

#Input initial values for the parameters as a list with named objects
inits <- list(beta0=betain[1],beta1=betain[2:(q+1)],gamma=rep(1,q),p=0.5)

#Use the arguments below for parallel processing, each chain runs on
#a different core
cl <- makePSOCKcluster(4)
jags.m <- parJagsModel(cl,name="para",file="C:\\yourdirectory\\ssvs.jag",
                       data = data,inits = inits, n.chains = n.chains)

#Define the quantities for which posterior distributions are required
params <- c("beta0","beta1","gamma")

#Use package coda to obtain the mcmc samples from the posterior distributions
samps<-parCodaSamples(cl, "para", params, n.iter=n.iter)
samps<-mcmc(samps,start=burn.in+1)
samps<-mcmc.list(samps)
parameter.mean <- summary(samps)[[1]][,1]

#Obtain the mean of the posterior distribution for the regression parameters
and selection probabilities for each predictor
beta=parameter.mean[1:(q+1)]
psel=parameter.mean[(q+2):(2*q+1)]

#Return estimates of the posterior means
return(list("beta"=beta,"psel"=psel))
}
```

S2. Sensitivity to standardisation of predictors

In the literature it is generally recommended to standardise all predictors (continuous and binary) to have mean zero and variance one, i.e. subtracting their means and dividing by their standard deviation ('1sd' method), so that the shrinkage applied is independent of the units. Gelman (2008) suggested centering the binary predictors and scaling the continuous predictors to have mean zero and standard deviation 0.5 (i.e. dividing by 2 standard deviations). This is the same as the standard deviation of a binary predictor with prevalence 0.5 ('2sd' method). Their reasoning was mainly that this yields more interpretable parameter estimates on a common scale.

A simulation study was carried out to investigate possible effects of these two types of standardisation in the terms of the predictive and variable selection performance of the shrinkage methods. In this study we considered ridge and two methods which perform variable selection, lasso and adaptive lasso, the second of which tends to apply more aggressive shrinkage,

 Copyright © 0000 John Wiley & Sons, Ltd.

i.e. it tends to shrink more coefficients to zero. The simulation study, in the same spirit of the simulation setting in Section 4 was as follows. Five binary (X_1-X_5) and continuous predictors (Z_1-Z_5) from a standard normal distribution were generated, along with the binary outcomes with prevalence 0.15 and 5 events per variable. Two of the binary predictors, X_1 and X_2, and two of the continuous predictors, Z_1 and Z_2 were given a null effect (true coefficient=0), while the rest had a non-zero effect size (true coefficient =0.5 on their original scale). Of the binary predictors, X_1 (noise predictor) and X_4 (true predictor) had prevalence approximately equal to 0.15 (i.e. they were 'unbalanced' predictors), whereas X_2 (noise predictor), X_3 and X_5 (true predictors) had prevalence approximately equal to 0.5 (i.e. they were 'balanced' predictors). We generated 1000 training datasets. For the variable selection performance we counted the proportion of simulations each predictor was selected in the model (Table S1) and for the predictive performance we calculated calibration, discrimination and root predictive mean square error on a validation dataset and presented their medians across 1000 simulations (Table S2).

As shown on Table S1 the 2sd standardisation resulted in differential selection between unbalanced binary predictors and continuous predictors/balanced binary predictors. Unbalanced binary predictors (noise and true) were less likely to be included in the selected model than the continuous or the balanced binary ones, both for lasso and adaptive lasso. In particular, for lasso, the 2sd approach resulted in false selection of the unbalanced binary noise predictor (X_1) in 34% of the simulations and the balanced noise one (X_2) in many more simulations, 51%. For the continuous noise predictor, the proportions were similar to the balanced one. For the true binary predictors, for lasso, the 2sd approach resulted in correct selection of the balanced binary predictors, X_3 and X_5, in 72% and 78% of the simulations, respectively, and of the unbalanced predictor (X_4) in 57%. For the 1sd standardisation this type of differential selection was much less pronounced; continuous noise predictors were falsely selected in similar proportion of simulations as the binary ones, balanced and unbalanced. Regarding the true predictors, for lasso, the unbalanced binary predictor (X_4) was less likely to be selected in the model, 65% of the simulations than the balanced ones X_3 and X_5, but the difference was less pronounced than the 2sd approach.

The predictive performance of the methods (also presenting results from ridge regression in Table S2) as assessed by calibration slope, C-statistic and predictive mean squared error, appeared to be very similar for the two methods of standardisation. When there are no strong unbalanced binary predictors, but there are noise unbalanced predictors the 2sd method is less likely to falsely select the noise binary unbalanced predictor than the 1sd. Conversely, when there exist strong unbalanced binary but no noise unbalanced binary predictors, then the 1sd method is more likely to correctly select the true unbalanced binary predictor than the 2sd method. In either case, the differences in terms of the predictive performance of the methods studied here, ridge, lasso, and adaptive lasso, were negligible (results not shown). In the simulations studies considered in the main paper we chose the first scaling pattern which is the most commonly used and the default option in most software packages. However, further research is necessary to assess the impact of standardisation in more general scenarios, especially when the number of noise predictors increases. Finally note that when the binary predictors were balanced, the predictive performance and the variable selection applied was exactly the same between the two standardisation methods, for lasso and adaptive lasso.

		Noise predictors				True predictors					
		Binary		Continuous		Binary			Continuous		
		$X1$	$\boldsymbol{X_2}$	Z_1	Z_2	X_3	$\boldsymbol{X_4}$	X_5	Z_3	Z_4	Z_5
1sd	LASSO	**0.48**	**0.50**	0.53	0.52	0.77	**0.65**	0.71	0.91	0.95	0.96
	ALASSO	0.28	0.28	0.30	0.28	0.64	0.49	0.55	0.87	0.91	0.95
2sd	LASSO	**0.51**	**0.34**	0.55	0.54	0.78	**0.57**	0.72	0.93	0.95	0.97
	ALASSO	0.30	0.23	0.31	0.30	0.67	0.46	0.56	0.87	0.92	0.96

Table S1. Proportion of simuations where each predictor was selected in the model for two types of standardisation. '1sd': centre and scale all predictors to have mean zero and variance one; '2sd' centre all predictors and scale continuous predictors to have variance 0.25. X_2 (noise) and X_5 (true) are binary predictors with low prevalence (0.15)

		Cal. slope	C-stat.	Root Pred. MSE
	MLE	0.67	0.694	0.0982
1sd	RIDGE	1.06	0.694	0.0830
	LASSO	0.96	0.692	0.0868
	ALASSO	0.86	0.690	0.0915
	MLE	0.67	0.694	0.0982
2sd	RIDGE	1.05	0.696	0.0824
	LASSO	0.95	0.692	0.0869
	ALASSO	0.85	0.690	0.0910

Table S2. Median calibration slope, C-statistic and root predictive mean square error over 500 simulations, for two types of standardisation of predictors. '1sd': centre and scale all predictors to have mean zero and variance one; '2sd' centre all predictors and scale continuous predictors to have variance 0.25

S3. Results from additional simulations

In this section we present results from the artificial simulations in section 5 and the Discussion. The scenarios considered were:

i. Scenario with noise predictors.
ii. Scenario with correlated predictors.

	X_1	X_2	X_3	X_4	X_5	X_6	X_7
MLE	1.00	1.00	1.00	1.00	1.00	1.00	1.00
BE	1.00	0.18	0.17	0.16	0.19	0.15	0.18
LSF	1.00	1.00	1.00	1.00	1.00	1.00	1.00
RIDGE	1.00	1.00	1.00	1.00	1.00	1.00	1.00
LASSO	1.00	0.29	0.27	0.26	0.28	0.25	0.29
ENET	1.00	0.40	0.39	0.36	0.40	0.38	0.40
ALASSO	1.00	0.16	0.14	0.13	0.17	0.12	0.17
SCAD	1.00	0.18	0.17	0.14	0.18	0.15	0.18
BLASSO	1.00	0.09	0.09	0.08	0.11	0.07	0.10
SSVS	1.00	0.08	0.08	0.09	0.11	0.08	0.09

Table S3. Model sparsity: Proportion of simulations each predictor was retained in the model (section 5.1) for EPV=3. X_1 is a true predictor and X_2-X_7 are noise predictors. Best performing method was SSVS with the fewest false positive selections.

 Copyright © 0000 John Wiley & Sons, Ltd.

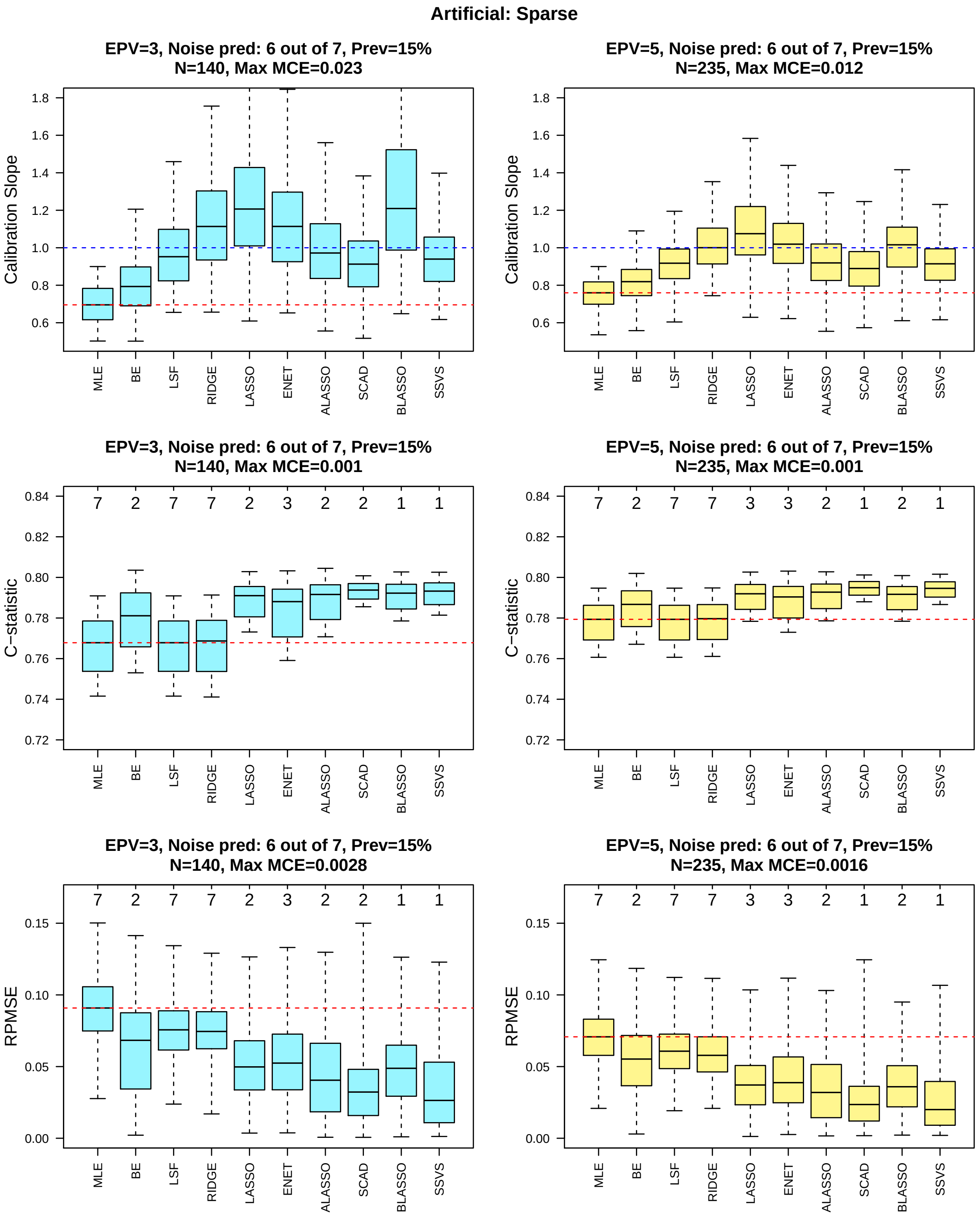

Figure S1. Model sparsity: performance measures for the sparse artificial scenario (section 5.1) for EPV=3 or 5 (6 out of 7 predictors are noise predictors). The number on top of each graph is the median number of predictors selected by each method. The red horizontal line is the median value for MLE. The blue horizontal line is the optimal calibration slope. Abbreviations: EPV: Events Per Variable, N=Number of observations, RPMSE: Root predictive Mean Squared Error, MLE: Maximum Likelihood Estimation, BE: Backwards Elimination, LSF: Linear Shrinkage Factor, ENET: Elastic Net, ALASSO: Adaptive Lasso, BLASSO: Bayesian Lasso, SSVS: Stochastic Search Variable Selection, MCE: Monte Carlo Simulation Error (for the median). The number of datasets (for each method) where the calibration slope could not be estimated for EPV=3 was: Lasso: 1; SCAD: 3; BLASSO: 7.

Copyright © 0000 John Wiley & Sons, Ltd.

Prepared using simauth.cls

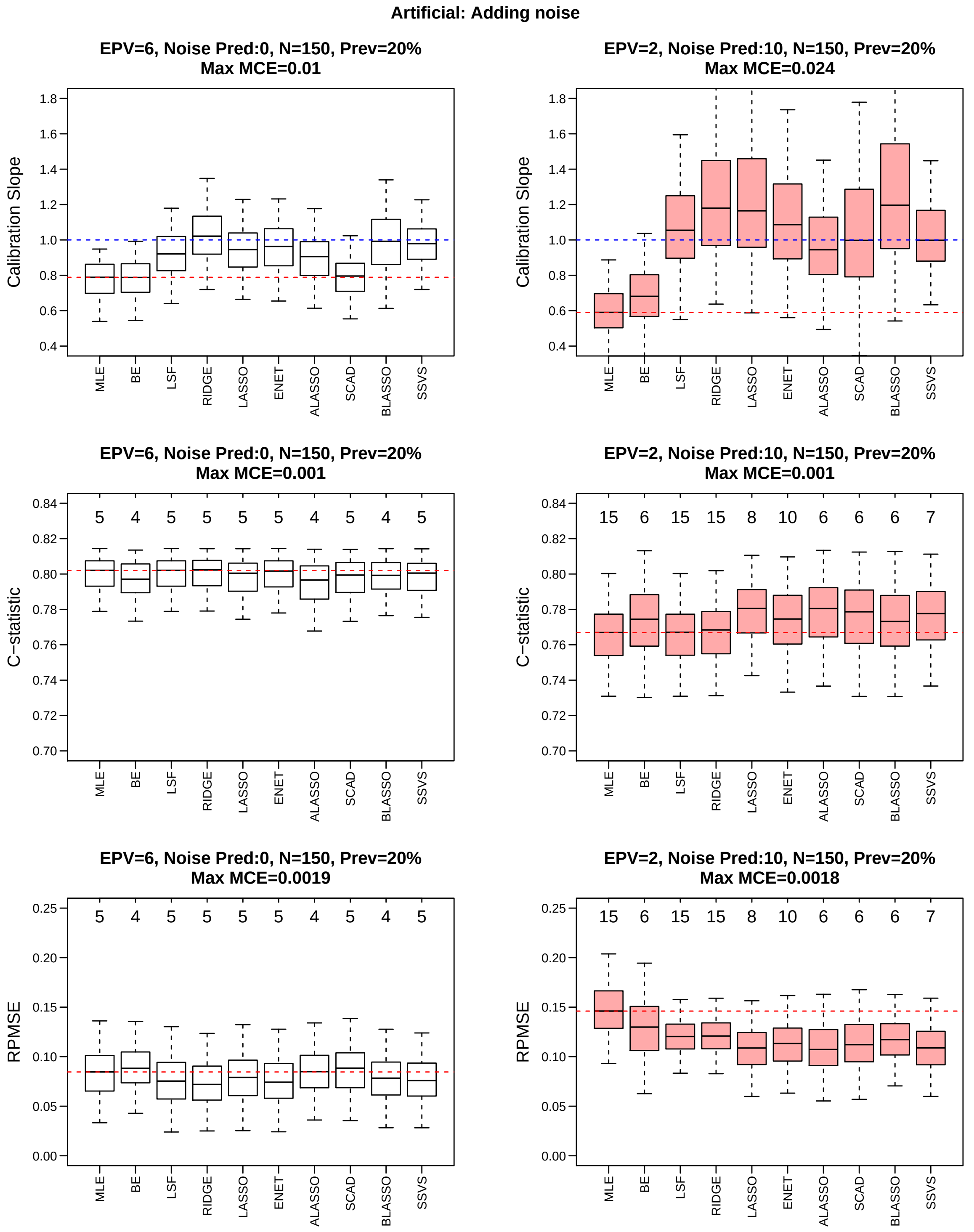

Figure S2. Addition of noise predictors: performance measures for the artificial scenario where 10 noise predictors are added to an existing model with five true predictors (section 5.1). The EPV for the initial model with five predictors is 6 while the the EPV after adding the noise predictors is 2. The number on top of each graph is the median number of predictors selected by each method. The red horizontal line is the median value for MLE. The blue horizontal line is the optimal calibration slope. Abbreviations: EPV: Events Per Variable, N=Number of observations, RPMSE: Root predictive Mean Squared Error, MLE: Maximum Likelihood Estimation, BE: Backwards Elimination, LSF: Linear Shrinkage Factor, ENET: Elastic Net, ALASSO: Adaptive Lasso, BLASSO: Bayesian Lasso, SSVS: Stochastic Search Variable Selection, MCE: Monte Carlo Simulation Error (for the median). The number of datasets (for each method) where the calibration slope could not be estimated for EPV=2 was: LSF: 10; Ridge: 3; Lasso: 8; ENET: 1; SCAD: 2; BLASSO: 9.

 Copyright © 0000 John Wiley & Sons, Ltd.

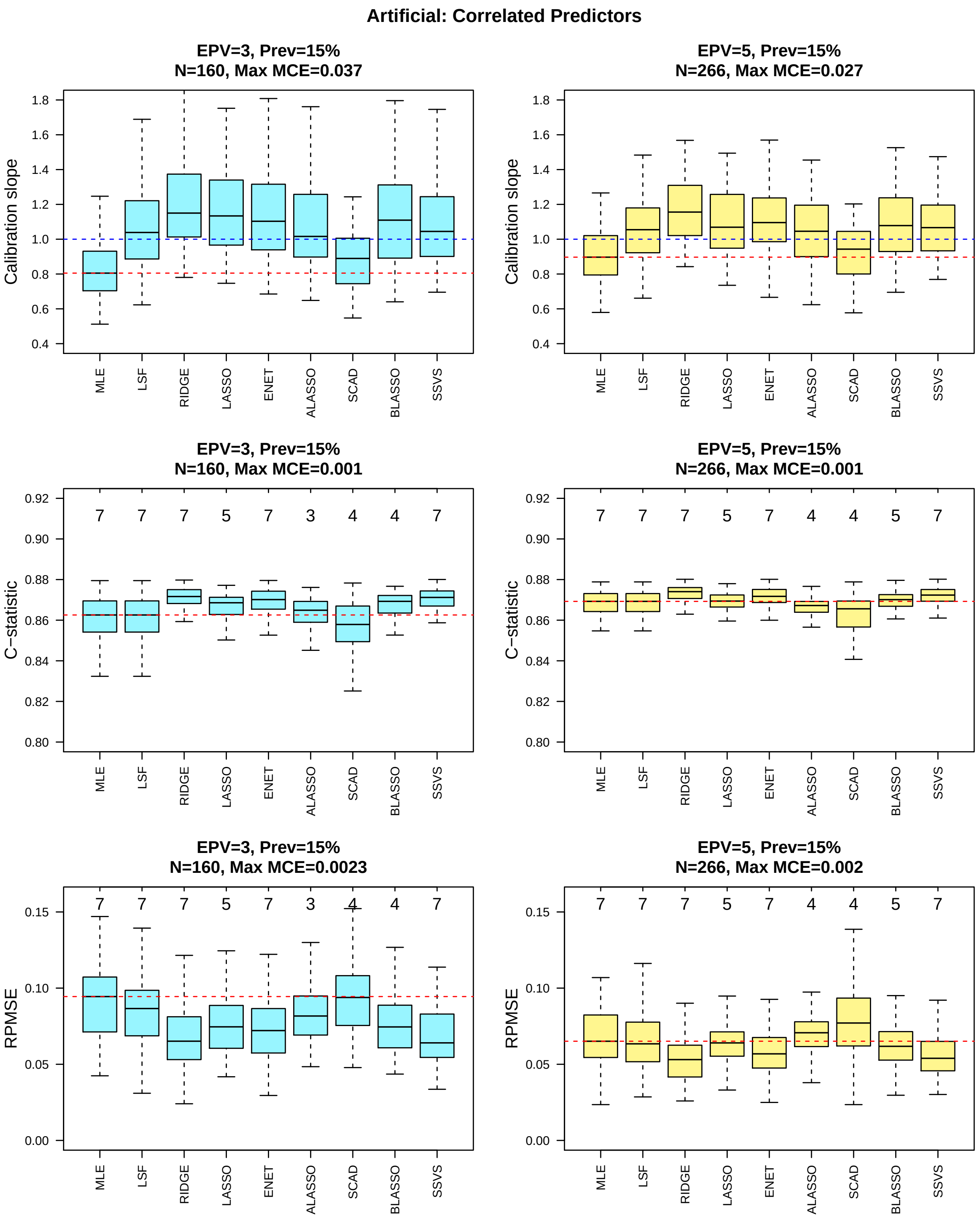

Figure S3. Correlated predictors: performance measures for the artificial scenario with correlated predictors (section 5.2) for EPV=3 or 5. The number on top of each graph is the median number of predictors selected by each method. The red horizontal line is the median value for MLE. The blue horizontal line is the optimal calibration slope. Abbreviations: EPV: Events Per Variable, N=Number of observations, RPMSE: Root predictive Mean Squared Error, MLE: Maximum Likelihood Estimation, BE: Backwards Elimination, LSF: Linear Shrinkage Factor, ENET: Elastic Net, ALASSO: Adaptive Lasso, BLASSO: Bayesian Lasso, SSVS: Stochastic Search Variable Selection, MCE: Monte Carlo Simulation Error (for the median). The number of datasets (for each method) where the calibration slope could not be estimated for EPV=3 was: SCAD: 2; BLASSO: 5.

Copyright © 0000 John Wiley & Sons, Ltd.

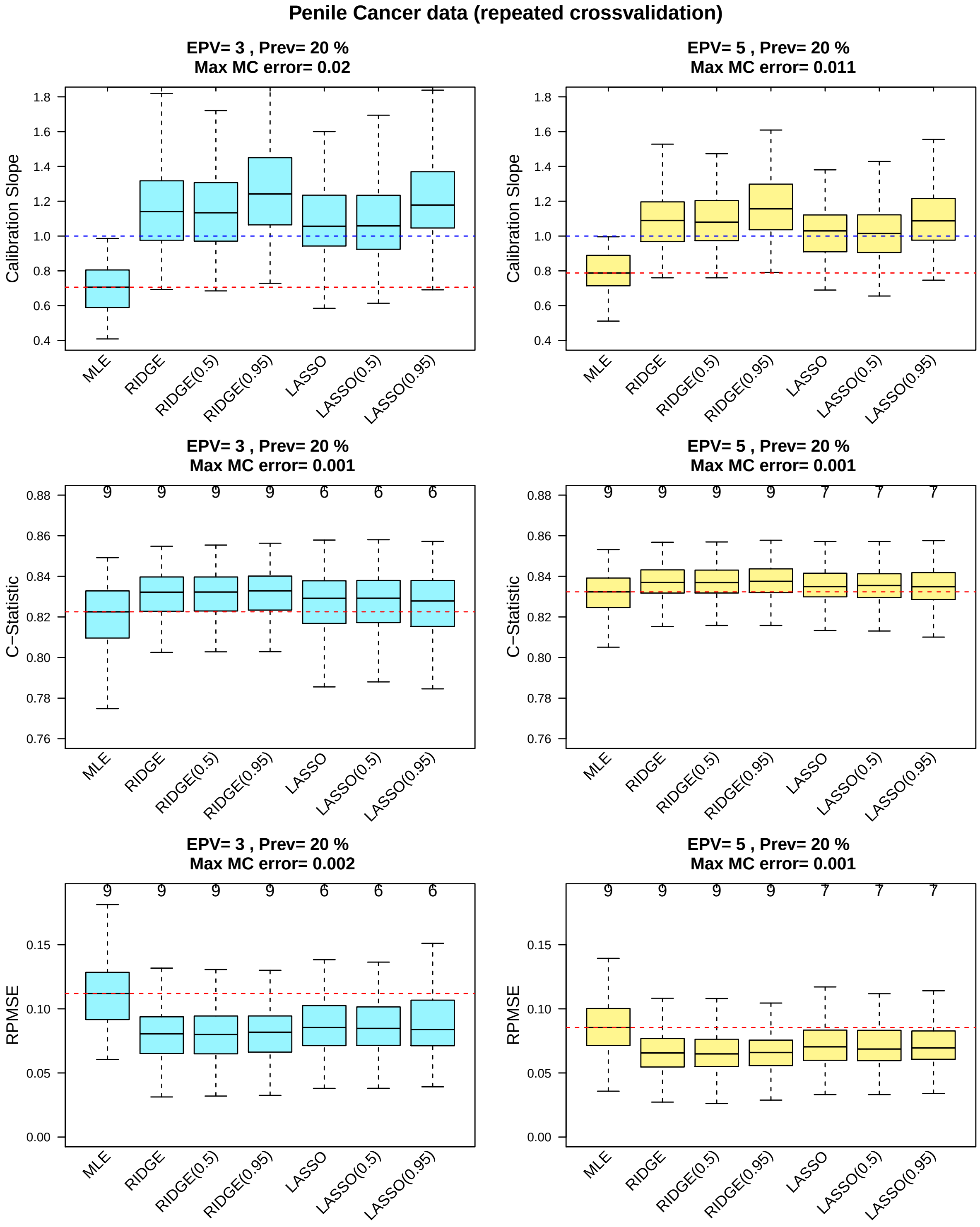

Figure S4. Performance measures for the the penile cancer example (section 4). Repeated cross-validation to assess sensitivity in the selection of tuning parameter. Ridge: Selection of tuning parameter was made using single crossvalidation. Ridge(θ): Selection of tuning parameter was made as the θ percentile (θ=0.5 or 0.95) of the optimal parameters over 50 cross-validations. Similarly for lasso.

 Copyright © 0000 John Wiley & Sons, Ltd.

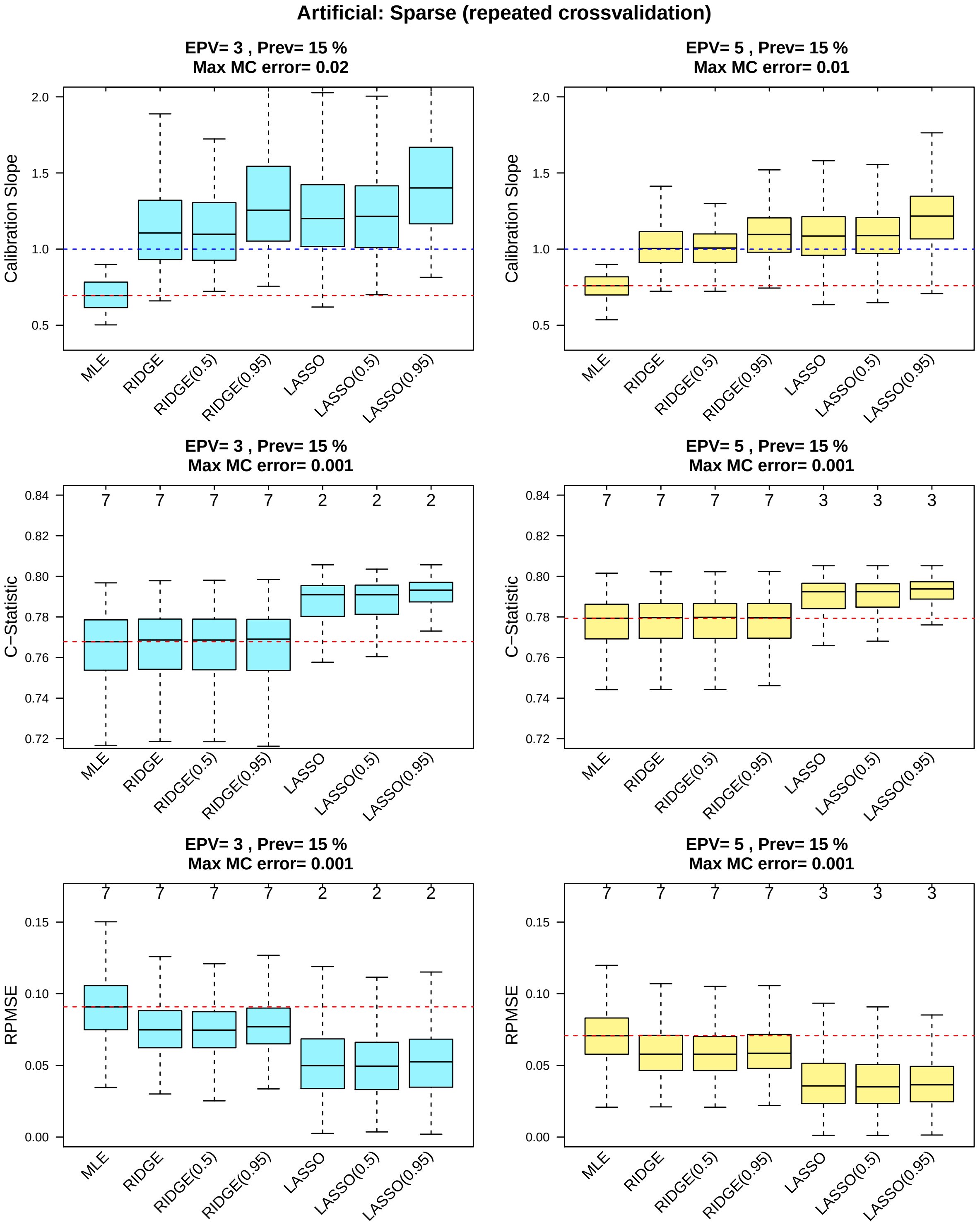

Figure S5. Model sparsity: performance measures for sparse artificial scenario (section 5.1) for EPV=3 or 5 (6 out of 7 predictors are noise predictors). Repeated cross-validation to assess sensitivity in the selection of tuning parameter. Ridge: Selection of tuning parameter was made using single cross-validation. Ridge(θ): Selection of tuning parameter was made as the θ percentile (θ=0.5 or 0.95) of the optimal parameters over 50 cross-validations. Similarly for lasso.
